# Hypoxia-induced HIF1α activation regulates small extracellular vesicle release in human embryonic kidney cells

**DOI:** 10.1038/s41598-022-05161-7

**Published:** 2022-01-27

**Authors:** Ana Muñiz-García, Montserrat Romero, Juan Manuel Falcόn-Perez, Patricia Murray, Antonio Zorzano, Silvia Mora

**Affiliations:** 1 Department of Molecular Physiology and Cell Signalling (Formerly Dpt. Cellular and Molecular Physiology), The University of Liverpool, Liverpool, L69 3BX UK; 2 Institute for Research in Biomedicine (IRB Barcelona), The Barcelona Institute of Science and Technology, Baldiri Reixac 13, 08028 Barcelona, Spain; 3 Department of Biochemistry and Molecular Biomedicine, Faculty of Biology, University of Barcelona, Av. Diagonal 643, 08028 Barcelona, Spain; 4 Exosomes Laboratory, Center for Cooperative Research in Biosciences (CIC bioGUNE), Basque Research and Technology Alliance (BRTA), 48160 Derio, Bizkaia Spain; 5 Centro de Investigación Biomédica en Red de Enfermedades Hepáticas y Digestivas (CIBERehd), Instituto de Salud Carlos III, 28029 Madrid, Spain; 6 IKERBASQUE, Basque Foundation for Science, 48015 Bilbao, Bizkaia Spain; 7 CIBER de Diabetes y Enfermedades Metabólicas Asociadas (CIBERDEM), Instituto de Salud Carlos III, Madrid, Spain; 8 Institute of Biomedicine, University of Barcelona, 08028 Barcelona, Spain

**Keywords:** Biochemistry, Transcription factors, Cell biology, Membrane trafficking

## Abstract

Extracellular vesicles (EVs) are membrane enclosures released by eukaryotic cells that carry bioactive molecules and serve to modulate biological responses in recipient cells. Both increased EV release and altered EV composition are associated with the development and progression of many pathologies including cancer. Hypoxia, a feature of rapidly growing solid tumours, increases the release of EVs. However, the molecular mechanisms remain unknown. The hypoxia inducible factors (HIFs) are transcription factors that act as major regulators of the cellular adaptations to hypoxia. Here, we investigated the requirement of HIF pathway activation for EV release in Human Embryonic Kidney Cells (HEK293). Time course experiments showed that EV release increased concomitantly with sustained HIF1α and HIF2α activation following the onset of hypoxia. shRNA mediated knock-down of HIF1α but not HIF2α abrogated the effect of hypoxia on EV release, suggesting HIF1α is involved in this process. However, stabilization of HIF proteins in normoxic conditions through: (i) heterologous expression of oxygen insensitive HIF1α or HIF2α mutants in normoxic cells or (ii) chemical inhibition of the prolyl hydroxylase 2 (PHD2) repressor protein, did not increase EV release, suggesting HIF activation alone is not sufficient for this process. Our findings suggest HIF1α plays an important role in the regulation of EV release during hypoxia in HEK293 cells, however other hypoxia triggered mechanisms likely contribute as stabilization of HIF1α alone in normoxia is not sufficient for EV release.

## Introduction

Extracellular vesicles (EVs) are membranous structures of different sizes released by cells, namely exosomes (50–200 nm in diameter), microvesicles (up to 1 µm) or apoptosomes (2 µm). Exosomes are formed and released within the endosomal membrane system as multi vesicular bodies (MVB). They are released to the extracellular space through fusion of these MVB with the plasma membrane^1,2^. These vesicles carry bioactive molecules (proteins, lipids and nucleic acids) and thus, participate in cell-to-cell communication^3–5^. The interest in EVs and particularly of small vesicles (exosomes) during the last decade has grown exponentially due to their therapeutic potential and use as biomarkers for human diseases^6–8^.

Low oxygen tension (hypoxia) is a feature associated with many pathophysiological conditions, including cancer malignancies, heart ischemia, stroke, obesity and kidney injury among others. The presence of hypoxia in solid tumours, is strongly associated with tumour growth, angiogenesis, malignant progression, metastasis and resistance to therapy^9^. Indeed hypoxia has been noted as a positive stimulus for exosome release in several cancer cells^10–12^ and in mesenchymal stromal cells^13^. In addition, we^14^ and others^15^ have described hypoxia-induced EV release in adipocyte cells, suggesting hypoxia may represent a universal stimulus that increases EV biogenesis and release. The precise molecular mechanisms that contribute to enhanced EV formation and release during hypoxic conditions however remain unknown.

The main machinery that regulates the transcriptional program underpinning the metabolic and cellular adaptations to hypoxia are the Hypoxia Inducible Factor (HIF) transcription factors. The active HIF transcription factors consist of two subunits: α and β^16^, however, in normal oxygen conditions, HIF-α is repressed by two oxygen dependent regulators: Prolyl Hydroxylases (PHD) and Factor Inhibiting HIF (FIH). Both hydroxylate HIF-α on proline and asparagine residues respectively, leading to its proteasomal degradation by the von Hippel-Lindau (VHL) protein^17^ thus, preventing the interaction with the constitutively expressed β subunit. When O_2_ levels decrease below 2%, both repressors lose their function enabling the stable expression of HIF-α, which then translocate to the nucleus, interact with HIF-β and together induce the transcription of several genes regulated by Hypoxia Response Elements (HRE)^18,19^. Three HIFα isoforms exist, HIF1, HIF2 and HIF3, with HIF1α being the most important and widely studied^20^.

In this study, we tested the hypothesis that activation of the HIF transcription factor pathway is directly responsible for the increase in EV release observed in hypoxic conditions. We evaluated whether shRNA-mediated knockdown of HIF isoforms during hypoxia or their stabilization in normoxic conditions enhanced EV release in human embryonic kidney cells. We stabilized HIF proteins through heterologous expression of oxygen insensitive HIFα mutants and through the blockage of the repressor pathway, via chemical inhibition of PHD. We found that HIF1α depletion but not HIF2α significantly reduced the number of EVs released in hypoxia. However, stabilization of HIF1α during normoxic conditions was not sufficient to enhance EV release. Our findings suggest that HIF1α activation along with additional cellular responsive mechanisms triggered by hypoxia, such as possibly those related to metabolic stress and autophagic/lysosomal activation flux contribute to the hypoxia-dependent EV release.

## Material and methods

### Reagents plasmids and antibodies

Tissue culture media and reagents (Dulbeco’s modified Eagle’s media, FBS, trypsin, penicillin/streptomycin, were from Sigma. PHD inhibitor IOX2 was from MedChem Express (cat. number: HY-15468). Plasmids encoding HIF1α P402A/P564A (*HA-HIF1alpha P402A/P564A-pcDNA3*) and HIF2α P405A/P531A (*HA-HIF2alpha-P405A/P531A-pcDNA3*) were from Addgene (cat. numbers: 18955 and 18956). CD63-GFP (CD63-pEGFP C2) and GFP (mEGFP-C1) were from Addgene (cat. numbers: 62964, and 54759). pKLpuro plasmids expressing validated shRNAs for HIF1α and HIF2α were obtained from Sigma-Merck (MISSION shRNAs library) and expressed as a pool of 4–5 shRNAs per each target gene Antibodies: anti-HIF1α was from BD Biosciences, (cat. number: 610958) or Cayman (cat. number: 000 6421) and used at 1:1000, anti-HIF2α was from Bethyl, UK (cat. number: A700-003) used at 1:2,000 or Santa Cruz (cat. number: Sc-13596) used at 1:500; anti-β-tubulin was from Merck-Sigma Aldrich, (cat. number: T4026) and used at 1:2000; anti-phospho-P70S6K(Thr389) was from Cell Signalling (cat. number: #9234) and used at 1:1000; anti-p62/SQSTM1 was from Progen (cat. number: GP62-C) and used at 1:1000; anti-phospho-eIF2α was from Abcam (cat. number: Ab32157) and used at 1:1000. For immunofluorescence studies antibodies against HIF1α and HIF2α were from Cayman and Santa Cruz respectively, (Cat. Numbers: 10006421 and Sc-13596) and used at 1:100).

### Cells and treatments

HEK293T (Human embryonic kidney 293 cells containing SV40 T-antigen), 3T3-L1 cells and NIH 3T3 cells were obtained from ATCC (catalogue numbers CRL-11268, CL-173 and CRL-1658 respectively). Human umbilical cord mesenchymal stromal cells (hUMSC) were obtained from NHS Blood and Transplant (NHSBT) at passage 2, the ventricular cardiomyocyte AC16 cell line was from Dr. A Planavila (University of Barcelona) and MBA-MD231 were from ECACC (catalogue n^o^: 92020424) AC16 cells were cultured as described^21^ and differentiated for 5 days before experiments. 3T3L1 adipocytes were cultured and differentiated as previously described^22^.All other cell lines were grown in DMEM high glucose (Sigma-Aldrich, D7777) supplemented with 10% fetal bovine serum (FBS; Sigma-Aldrich, F7524) and 1% penicillin–streptomycin (P/S; Sigma-Aldrich, P0781) at a regular humidified incubator (37 °C, 5% CO_2_). For EV analysis, prior to each experiment, cell conditioned media was removed, washed in PBS and replenished with DMEM supplemented with 2% exosome free FBS, and 1% penicillin/streptomycin. Hypoxic conditions were obtained using a H35 hypoxia workstation (Don Whitley Scientific, UK) at 1% O_2_ v/v, 5% CO_2_ v/v and 94% N_2_ v/v at 37 °C. Plasmid transfections: HEK293T cells were plated in 6 or 12-well dishes and transfected with 0.5 µg-2 ug of DNA with Lipofectamine 3000 Transfection Reagent (Life Technologies) following the manufacturer’s instructions. Media was removed and replenishedwith DMEM containing exosome free 2% FBS at 2 h post-transfection. For EV analysis media was collected at the different time points as indicated in the figure legends. For the HIF Knock down experiments, cells were transfected with a pool of 5 plasmids expressing validated shRNAs (Sigma MISSION shRNA Library) targeting either HIF1α or HIF2α or both isoforms together. Transfected cells were selected with puromycin at 2 µg/ml for 72 h. Cells were then washed in PBS and the media changed to DMEM containing 2% exosome-free FBS prior to the hypoxia/normoxia exposure for 20 h.

### Isolation of extracellular vesicles and analysis

Extracellular vesicles (EVs) were either isolated from conditioned media using differential centrifugation as we previously described^14,23^. In some experiments (indicated in the figure legends) EV content was measured directly from the conditioned media following two consecutive centrifugations: at 2000×*g* for 10 min and 10,000×*g* for 30 min, to discard intact cells, cellular debris and larger EVs respectively. EV analysis was carried out by nanoparticle tracking analysis (NTA) using a Nanosight NS300 instrument (Malvern, UK). All samples were analysed obtaining 3–5 captions of 60 s each. The mean was used for their analysis. NTA data was normalized taking into account the total amount of EVs and normalized to the protein content in whole cell lysates determined by Bradford or BCA method. All size distribution graphs from the NTA EV data shown in the main text can be found in the Supplementary Figs. S1, S2, S5 and S6. For tetraspanin protein marker detection in EVs, the samples were analysed using the ExoView platform with the Tetraspanin Kit on ExoView R200 system (NanoView Biosciences, UK) following the manufacturer instructions.

### Cryo-electron microscopy

Extracellular vesicle preparations directly absorbed onto carbon grids was carried out as we previously described^14^. Images were taken using a JEM‐2200FS/CR transmission cryo‐electron microscope (JEOL, Japan).

### Cellular lysates and immunoblotting

Cellular lysates were freshly obtained at the end of each experiment using a HEPES lysis buffer (50 mM HEPES pH 7.5, 150 mM NaCl, 10 mM EDTA, 10 mM Na_4_P_2_O_7_, 10% Glycerol, 2 mM Na-vanadate, 100 mM NaF, 1% Triton X-100, 500 μM PMSF, 2 μM Pepstatin, 1 μM Leupeptin, 10 μM Aprotinin). Cellular lysates from hypoxic cells were obtained inside the hypoxia workstation with previously equilibrated PBS and lysis buffer to prevent re-oxygenation. Protein samples were prepared in Laemmli Sample Buffer resolved on SDS-PAGE, transferred to nitrocellulose membranes, blotted in 5% non-fat milk in Tris-buffered saline (pH 7.6) and subsequently immunoblotted with primary antibodies and fluorescent labelled secondary antibodies (LI-COR) at the dilutions recommended by the manufacturer. Membranes were washed in Tris Buffered Saline containing 0.1% Tween and visualized in a LI-COR Odyssey imaging system (LICOR biosciences, UK). Quantification of blots relative to reference protein as indicated in the figure legends was carried out using image J (NIH).

### Cell immunostaining and imaging

Cells were fixed and immunostained as previously described^22^. Briefly, cells were washed twice with PBS and fixed with 2% paraformaldehyde/PBS for 15 min. Following fixation, cells were washed in PBS and permeabilised with PBS containing 0.2% Triton X-100 for 10 min. Cells were washed again and blocked in TBS containing 1% BSA, 5% Donkey serum (Sigma-Aldrich, D9663) and 0.1% Tween. Cells were washed once with TBS with 0.1% Tween (TBS-T) and incubated with the primary antibodies against HIF1α (Cayman, #10006421) and HIF2α (Santa Cruz, Sc-13596) diluted 1:100 in 1% BSA TBS-T for 1 h and 30 min. Unbound primary antibody was washed in TBS-Tween 0,1% and incubated with fluorescently labelled secondary antibodies (Invitrogen, A21206 and A10037) at 1:1000 for 1 h and 30 min. Lastly, cells were again washed three times with TBS-T, and coverslips were mounted on glass slides using Prolong Gold Antifade Mountant with DAPI (Invitrogen, P36935). Images were acquired with a Zeiss AxioObserver inverted fluorescence microscope with Apotome2 and Zeiss ZenPro software.

### mRNA expression

Total RNA was extracted using the Trizol reagent (LifeTechnologies) or RNeasy (Qiagen) and following the manufacturer protocol. RNA was resuspended in water and stored at – 80 °C until used. 1–2 ug of total RNA was used for cDNA synthesis using the High-Capacity RNA-to-cDNA Kit (Applied Biosystems) or SuperScript III Reverse Transcriptase (Invitrogen). Real time PCR was performed using the TaqMan protocol or SYBR Green method (see below) and relative mRNA expression levels were calculated using the 2ΔΔCt method^24^ with the *18S or ACTB* as reference genes^24^. The TaqMan Assay IDs were: *18S* (Hs99999901_s1); *HIF1A* (Hs00153153_m1); *EPAS1* [alias HIF2A] (Hs01026149_m1); *SLC2A1* [GLUT1 expressing gene] (Hs00892681_m1); and *VEGFA* (Hs00900055_m1). -For SYBR Green, Primers 5′–3′ were: for *VEGFA*: Forward: CTTGCCTTGCTGCTACCT Reverse: GCAGTAGCTGCGCTGATAGA; *SLC2A1* (GLUT-1): Forward : GGCTTCTCCAACTGGACCT, Reverse: CCGGAAGCGATCTCATCGAA; *VEGFB* Forward: GGGCACACACTCCAGGCCATC; Reverse: CCTTGACTGTGGAGCTCATGGGCC; *ACTB* Forward: GATGAGATTGGCATGGCTTT; Reverse: CACCTTCACCGTTCCAGTTT.

### Statistical analysis

Analysis of variance was carried out using GraphPad Prism6, setting a confidence interval of 95% and p < 0.05 as a statistical significance value.

## Results

### Hypoxia increases small extracellular vesicle release in HEK293 cells concomitantly with a rapid activation of HIF pathway

**Figure 1:**
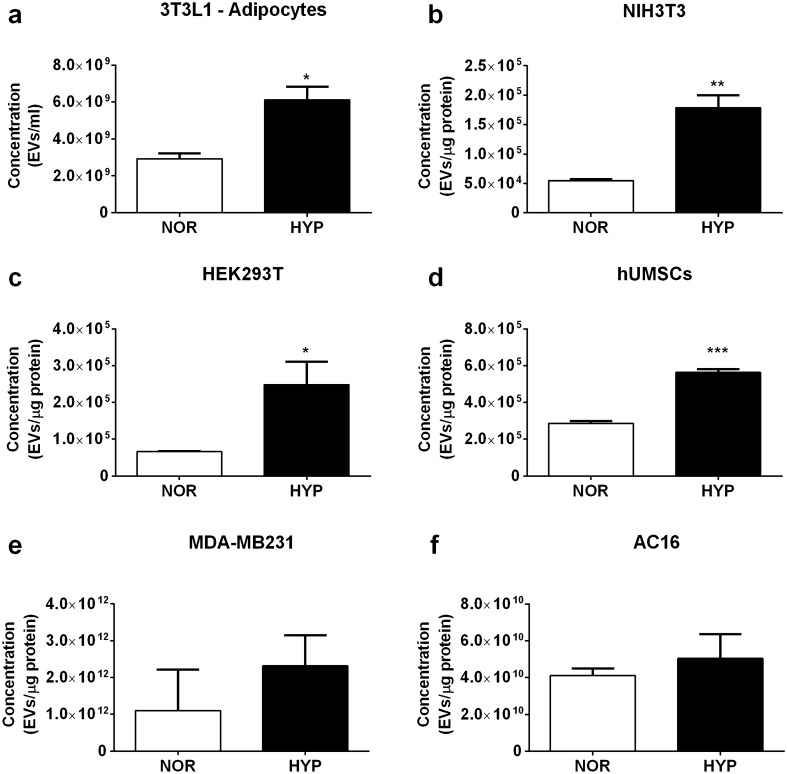
Hypoxia enhances small EV release in several cell lines. Several cell types including 3T3L1 adipocytes, NIH-3T3 fibroblasts, HEK293T, hUMSCs, AC16 and MDA-MB231were cultured and differentiated as indicated in methods section. Cells were washed in PBS and media changed to serum free or supplemented with 2–5% of exosome-free serum. Cells were exposed to hypoxia for 24 h with the exception of AC16 cells which were treated for 16 h and hUMSCs for 2h. Control cells were kept in normoxia for the same amount of time. The conditioned media was collected and small EVs were isolated by differential ultracentrifugation and quantified by NTA analysis. Data shows the mean ± standard error of the mean (SEM) of N = 3–4 independent experiments. Data were normalized to the protein content in the cellular lysates. Statistical Analysis two tailed T-test. *indicates < 0.05, **p < 0.01, ***p < 0.001. *NOR* normoxia, *HYP* hypoxia.

We previously reported an increase in the release of small extracellular vesicles (exosomes) in cultured 3T3L1 adipocytes subjected to 24 h of hypoxic conditions^14^. In order to further investigate the underpinning molecular mechanisms of this response, we first evaluated the effects of 1% O_2_ on small EV release in a range of cell types to ascertain the universality of this response. Alongside the 3T3L1 adipocytes, used here as positive controls, we tested NIH-3T3 fibroblasts, human embryonic kidney cells (HEK293T), a ventricular cardiomyocyte cell line (AC16), a breast cancer cell line (MDA-MB231) and human umbilical mesenchymal stromal cells (hUMSCs). Equal amounts of cells were plated and either cultured in normoxic (5% CO2, 95% air) or hypoxic conditions (1% Oxygen, 5% CO_2_ and 94% N_2_ v/v) for up to 24 h, and EV release into the conditioned media was analysed by NTA analysis and normalized to the amount of protein in cellular lysates. While the tolerance to hypoxia was specific to each cell line, most cells showed a robust increase in EV release upon the hypoxic conditions tested (Fig. 1).

**Figure 2:**
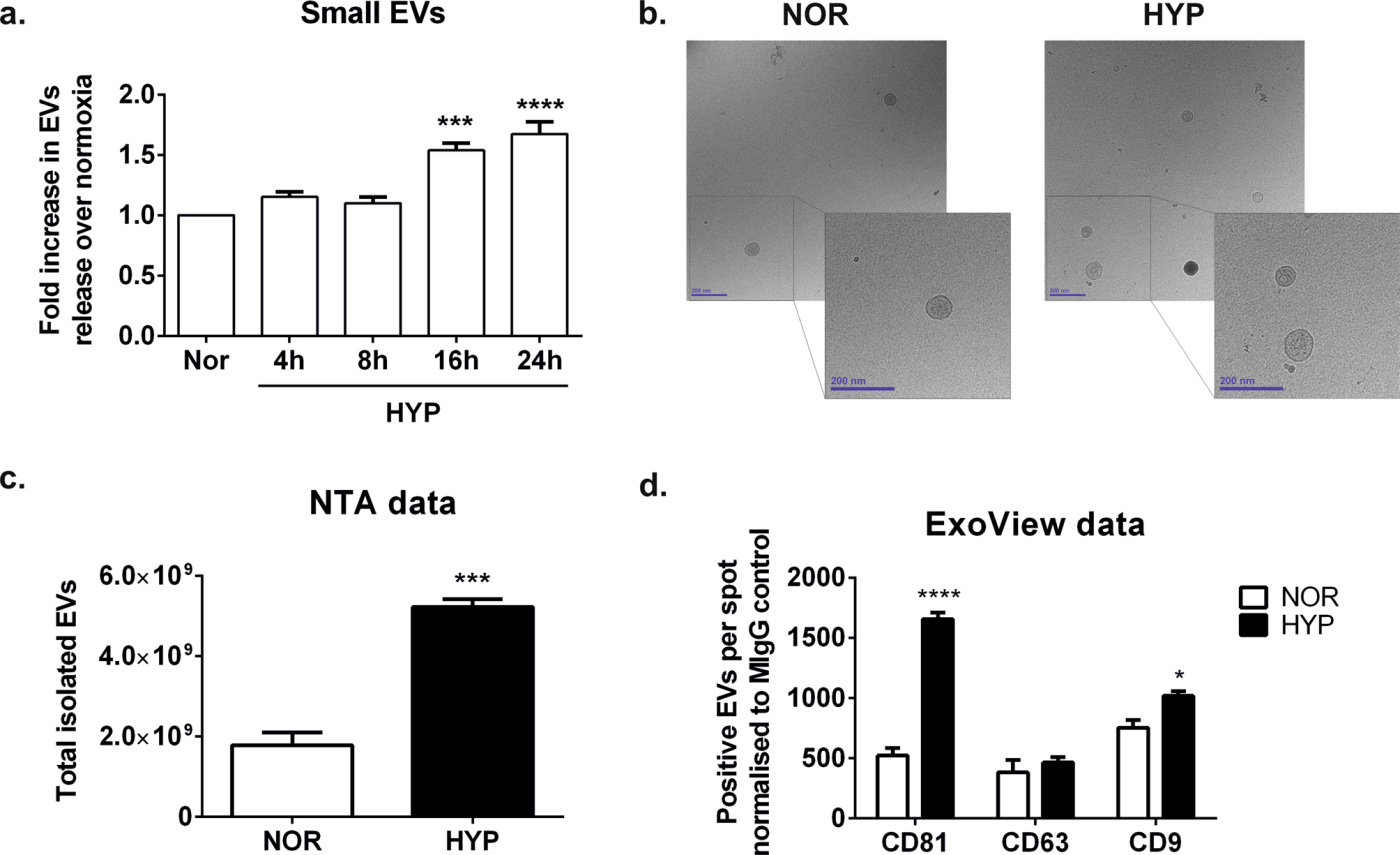
Steady increase of small EV release under hypoxic conditions in HEK293T cells. (**a**) Time course experiment of EV release in HEK293T cells subject to hypoxia. The graph shows the NTA quantification of small EVs isolated by differential centrifugation protocol after a ultracentrifugation step at 100,000×*g* 75 min. Each data point was corrected by protein content in the cellular lysates and relativized to EVs determined in normoxic cells at the same time point. Data combined from N = 4 experiments, graphs shows mean ± SEM. Statistical analysis: One Way ANOVA with Dunnett’s multiple comparisons test. **p < 0.01, ***p < 0.001. (**b**) Cryoelectron micrographs of small EVs isolated from normoxic and hypoxic cells at 16 h time point. Magnification: 30,000 ×. Scale bar: 200 nm. (**c**) Nanoparticle tracking analysis quantification of small EVs from the conditioned media of normoxic or hypoxic cells. (**d**) ExoView characterization of EVs. Analysis of exosomal markers was carried out with the ExoView Tetraspanin Kit following the manufacturers protocol (**c**) and (**d**). Graphs show data analysed in triplicate (n = 3). Stats: (**c**) Two tailed t-test and (**d**) Two-way ANOVA, followed by Sidak’s multiple comparisons test, where * indicates p < 0.05, ***p < 0.001 and ****p < 0.0001.

We next sought to investigate whether this response is due to the activation of the HIF transcription factors. We chose the HEK293T cell line for these studies since these cells responded well to hypoxic conditions increasing small EV release. We first compared the kinetics of release of small EVs into the media, to the kinetics of HIF stabilisation by performing a time course analysis in 1% O_2_. We observed a steady increase in the amount of small EVs present in the conditioned media, which was significantly enhanced from 16 h of exposure to hypoxia detected by NTA both following differential centrifugation (Fig. 2a) and when measured directly from the conditioned medium (Supplementary Fig. S5) in independent experiments. Cryo-electron microscopy analysis (Fig. 2b) and NTA analysis (Fig. 2a,c) of the released vesicles and size distribution analysis by NTA (Supplementary Fig. S2) confirmed the presence of small EVs consistent with exosomes. Analysis of the vesicles by Exoview or western blot analysis confirmed an enrichment in standard EV marker tetraspanin proteins (Fig. 2d and Supplementary Fig. S3).

**Figure 3:**
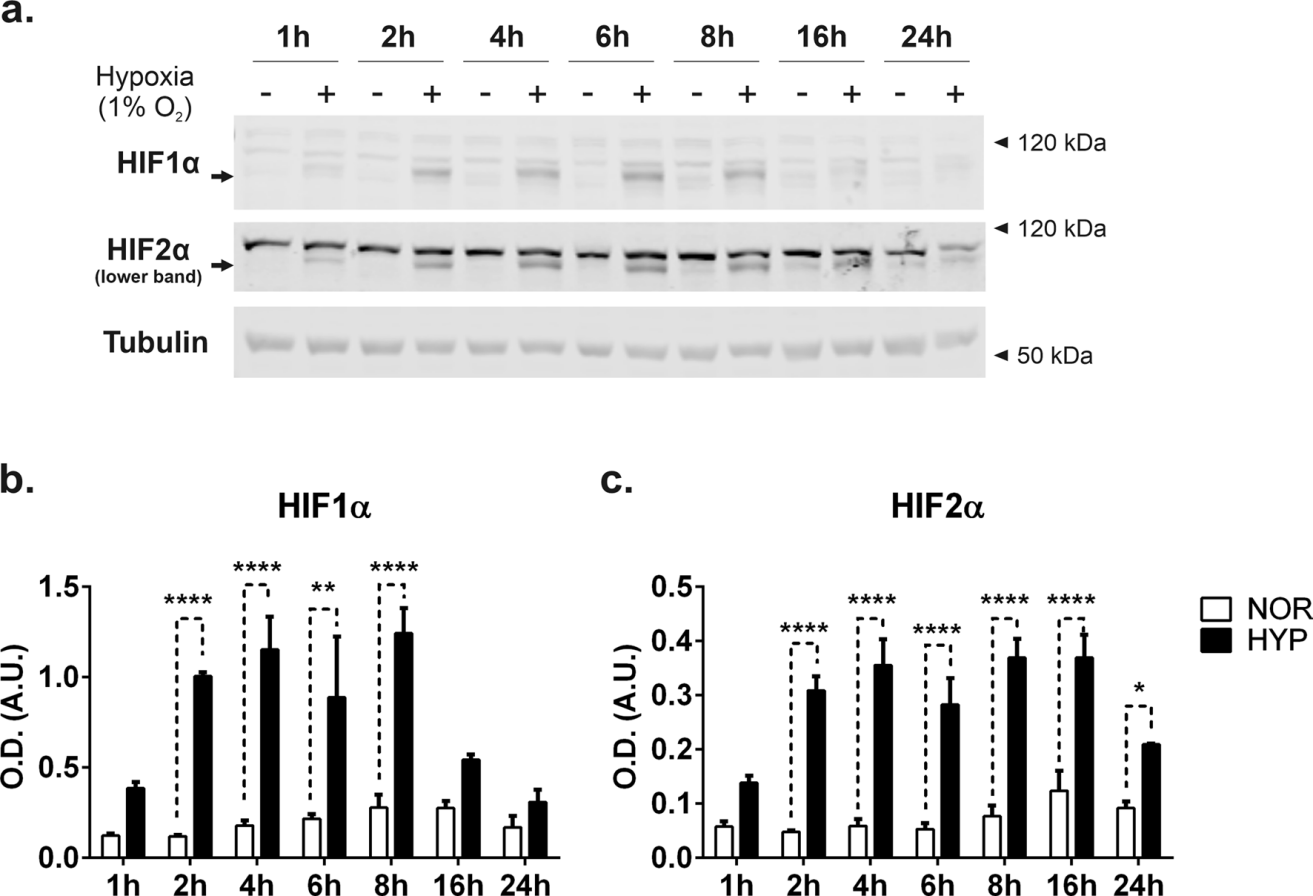
HIF pathway dynamics in HEK293T exposed to hypoxia. (**a**) Cells were exposed to normoxic or hypoxic conditions for the times indicated. Lysates were obtained as indicated in the methods loaded onto SDS-PAGE and immunoblotted with antibodies for HIF1α or HIF2α. Note the upper bands are non-specific and commonly detected by this^26^ (and other^27,28^) commercial antibodies. Immunodetection of β-tubulin was used as loading control as indicated. Blots show a representative experiment of n = 3. (**b**, **c**) Quantification of western blot analysis. Graphs show the mean ± SEM. Statistical analysis: Two-way ANOVA, followed by Sidak’s multiple comparisons test. *indicates < 0.05, **p < 0.01, ***p < 0.001.

In parallel, we examined the levels of HIF1α and HIF2α isoforms by western blot analysis (Fig. 3a,b). We observed a rapid stabilization of both isoforms, peaking at 8 h for HIF-1α and stable up to 16 h for HIF-2α (Fig. 3a–c). The levels of both isoforms declining after 24 h is consistent with previous studies^25^.

### Knock down of HIF1α but not HIF2α abrogates EV release in HEK293T cells upon hypoxic exposure

**Figure 4:**
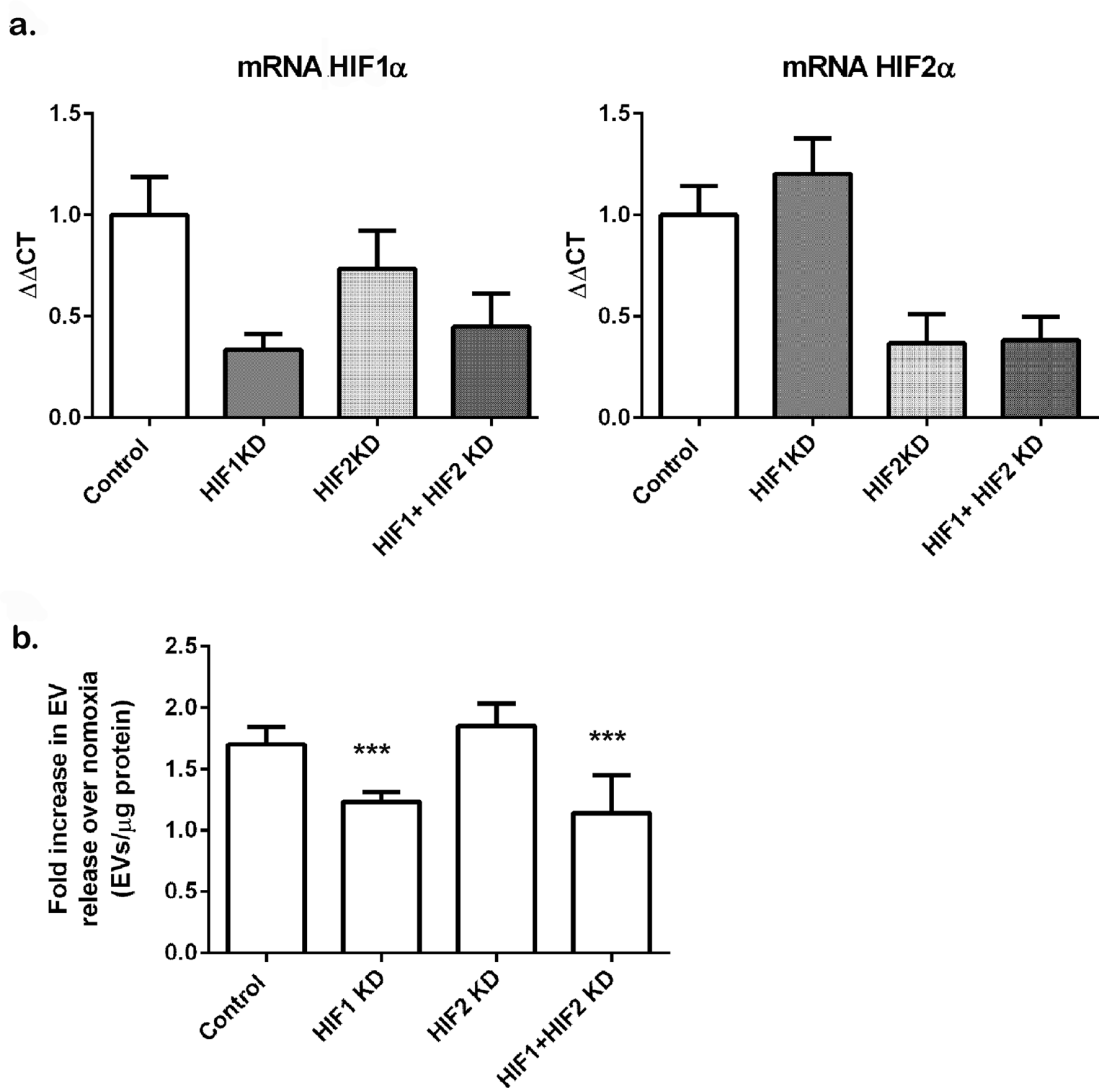
Knockdown of HIF1α reduces EV release in hypoxic HEK293T cells. Cells were transfected with plasmids encoding for validated shRNAs specifics for HIF1α, HIF2α or both as detailed in the methods section. Following selection of transfectants in puromycin, cells were exposed to normoxic or hypoxic conditions for 16 h. (**a**) Expression of HIF1 and HIF2 by real time PCR. Total RNA was isolated from cells and mRNA abundance for HIF1α or HIF2α was determined by real time PCR, Data is mean ± SDEV of a representative experiment, of N = 4 experiments. (**b**) EVs were isolated by differential centrifugation protocol after an ultracentrifugation step at 100,000×*g* for 75 min to pellet EVs. EVs were analysed by NTA. Data shows mean ± SEM of N = 3–4 experiments per group. EVs were normalized to total protein content in the cellular lysates and referred to the normoxic group to combine the data from different experiments. Statistical analysis One Way ANOVA: ***indicates p ≤ 0.05.

To determine whether the increase in EV release in hypoxic conditions is dependent on the activation of HIFs proteins, we depleted the expression of HIF1α or HIF2α individually or in combination by transient transfection in HEK293T cells. For these experiments we transfected cells with a pool of 4–5 validated plasmids encoding for shRNAs targeting each of these genes. Following the selection of transfected cells in puromycin, cells were then incubated in normoxic or hypoxic conditions (1% O_2_) for 16 h in EV-free media. Conditioned media was isolated and small EVs purified by differential centrifugation and subjected to NTA analysis. Knock down efficiencies as assessed by real time PCR were in the range of 70–83% for HIF1α depletion and 46–82% for HIF2α Knock down. Depletion of both isoforms together achieved a depletion of 46–71% for HIF1 and 64–77% for HIF2 in all the experiments performed. A representative experiment is shown in Fig. 4a. To analyse the EV release data we combined experiments that achieved a similar knockdown efficacy for each isoform, and expressed the results as EVs normalized by protein in the cellular lysate as relative to the EV release in the normoxic condition for each experiment. The data revealed that knockdown of HIF1α but not HIF2α impaired the increase in EV released in hypoxic cells (Fig. 4b).

### Expression of oxygen insensitive HIF1α and HIF2α stable mutants does not increase small EV release in normoxic conditions

To confirm these data and to evaluate whether HIF1α activation is sufficient to increase EV release in these cells, we next sought to mimic HIF pathway activation, in normoxic conditions. To this end, we transiently transfected cells with plasmids expressing oxygen resistant forms of HIF proteins, whereby the two proline residues that become hydroxylated under oxygen conditions are mutated to alanine, thus conferring the HIF proteins’ stability under normal ambient oxygen concentrations. We expressed HIF1α P402A/P564A and HIF2α P405A/P531A, either singly or in combination in HEK293T cells and compared the release of small EVs into the conditioned media to that of untransfected cells.

Since transfection experiments are usually done in much smaller plate format with a reduced volume of conditioned media, we first sought to validate the measurement of EVs directly from the conditioned media of cultured cells in transfected cells, following a low speed (2000×*g*) centrifugation to eliminate intact cells and large debris, and a 10,000×*g* centrifugation to eliminate organelles and large vesicles. We also aimed to rule out any possibility that a transient transfection with a lipid reagent (i.e. Lipofectamine 3000) could interfere with the measurement of EVs in this way. For this reason, we first carried out a validation experiment in which cells were transiently transfected with a plasmid coding for EGFP-tagged CD63 following the manufacturer’s standard protocol. 24 h following the transfection EV content in the conditioning media was measured from the cells expressing a GFP-CD63 protein and compared it to cells expressing only GFP or to untransfected cells as controls. As expected, cells expressing the tetraspanin CD63-GFP which is present in extracellular vesicles showed increase in EV release (Supplementary Fig. S4) whereas no increase in EVs content was detected in control untransfected cells or cells expressing only the GFP protein. The EVs directly measured from the conditioned media by NTA show a clean peak at sizes 50–200 nm, consistent with the detection of small vesicles (exosomes) (Supplementary Fig. S4). Thus, this experiment served to validate our EV detection method directly in the small volumes of conditioned media obtained from cells, following a low speed and a 10,000×*g* centrifugation without the need for ultracentrifugation. This experiment also dismissed any potential interference of the transfection method with the measurement and detection of EV in the conditioned media.

**Figure 5:**
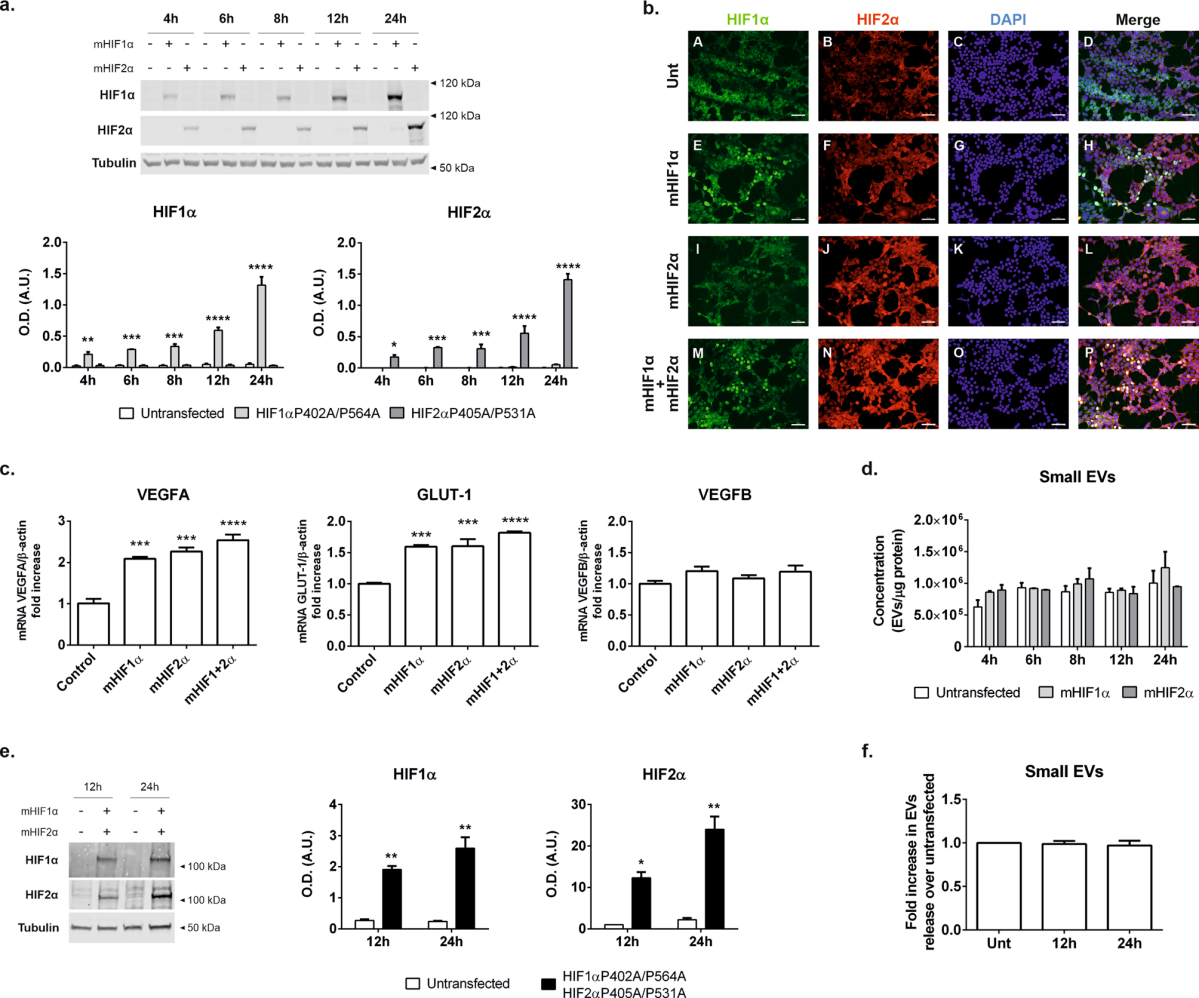
Overexpression of oxygen stable HIF mutant proteins does not increase small EV release in normoxic HEK293T cells. HEK293T cells were transiently transfected with plasmids coding for normoxia-stable HIF1α P402A/P564A (mHIF1α) or HIF2α P405A/P531A (mHIF2α) mutants as indicated in the methods section. (**a**) Immunoblot of cellular lysates. Cell homogenates were resolved in SDS-PAGE and immunoblotted with antibodies for HIF1α, HIF2α or  β-tubulin. Representative western blot analysis is shown on top and quantification of immunoblots carried out in Image J (NIH) is shown underneath. Graphs show the mean ± SEM of a representative experiment of N = 2 experiments performed each with two biological replicates. Statistical analysis: Two-way ANOVA, followed by Sidak’s multiple comparisons test. **p < 0.01, ***p < 0.001. (**b**) Immunolocalization of mHIF1α and mHIF2α in HEK293T cells. Following 24 h after transfection cells were fixed, permeabilized and stained with antibodies against HIF1α and HIF2α as described in the methods section. Nuclei were stained with DAPI. (scale bar = 50 µm). (**c**) mHIF1α and mHIF2α expressing cells show induction of mRNAs of hypoxia activated genes. Total RNA was isolated from cells transfected with mHIF1α and mHIF2α expressing plasmids. Following RNA extraction a RT-qPCR was performed to analyse the transcription levels of genes known to be highly upregulated by HIF (*VEGFA* and *SLC2A1*) and a third one known for being generally stable and not affected by HIF transcriptional program (*VEGFB*) as control. Graphs show the expression levels calculated as the 2^−ΔΔCt^ values for each gene normalised to β-actin. Graphs show mean ± SEM of n = 3 replicates, each analysed in duplicate. Statistical analysis: One-way ANOVA, *** indicates p < 0.001. (**d**) Quantitation of small EVs by measurement of vesicles in the supernatant of the 10,000×*g* centrifugation of the conditioned media. Samples were of untransfected cells or from cells overexpressing either mHIF1α or mHIF2α. Data was normalized to the amount of total protein content present in the cellular lysate. Graph shows the mean ± SEM of a representative experiment of N = 2 experiments performed each with two biological replicates. (**e**) Immunoblot of cells expressing both mHIF1α and mHIF2α. Cellular lysates obtained from untransfected cells or cells expressing both mHIF1α and mHIF2α were resolved by SDS-PAGE and immunoblotted with antibodies againts HIF1α, HIF2α or β-tubulin. A representative western blot analysis is shown on the left. The graphs on the right show the quantification data (mean ± SEM) of N = 4 experiments each performed in duplicate. Statistical analysis: Two-way ANOVA, followed by Sidak’s multiple comparisons test. * p < 0.05. (**f**) Quantitation of small EVs by measurement of vesicles in the supernatant of the 10,000×*g* centrifugation of the conditioned media. Samples were of untransfected cells or from cells overexpressing mHIF1α and mHIF2α. Data was normalized to the amount of total protein content in the cellular lysate. Graph shows the mean ± SEM of N = 4 experiments performed each in duplicate.

Transfection of plasmids encoding either HIF mutant in HEK293T achieved a rapid expression of HIF proteins that increased steadily over a period of time achieving a robust expression of protein levels as determined by western blot analysis (Fig. 5a). Protein levels were also detected by immunofluorescence (Fig. 5b). To ascertain that the mutant HIF proteins expressed were transcriptionally active we determined the mRNA abundance of *SLC2A1* (which expresses the glucose transporter GLUT1) and *VEGFA* which expresses the vascular endothelial growth factor, both genes are upregulated during hypoxia and are known to be regulated by HIFs. Additionally we measured the mRNA abundance of *VEGFB* which is not activated by HIFs transcriptional activity. We detected a higher expression levels of SLC2A1 and VEGFA specifically in cells that had been transfected with either mHIF1α and mHIF2α alone or in combination, compared to control cells whereas no effect was seen in the expression of VEGFB as expected (Fig. 5c). However, there was no difference in the amount of small EVs detected in the conditioned media following the single expression of either mutant HIF isoform compared to the untransfected control cells (Fig. 5d).

Since these data raised the possibility that the lack of induction of EV release could be due to the need for both HIFα isoforms being stabilized, as occurs under normal hypoxic conditions, we repeated the experiment co-expressing both HIF1α and HIF2α mutant proteins. Again, we observed a strong stabilization of both HIFα isoforms 12 and 24 h after transfection (Fig. 5e). Despite this, we detected no effect on EV release (Fig. 5f).

### Chemical inhibition of PHD2 does not affect small EV release in normoxic conditions

To confirm our observation with the HIF stable mutants, we sought to inhibit HIF degradation through the chemical inhibition of the Prolyl Hydroxylase 2 (PHD2). To this end, we treated cells with IOX2, a specific potent chemical inhibitor that has previously been established to mimic the hypoxia effects on the HIF pathway^29^.

**Figure 6:**
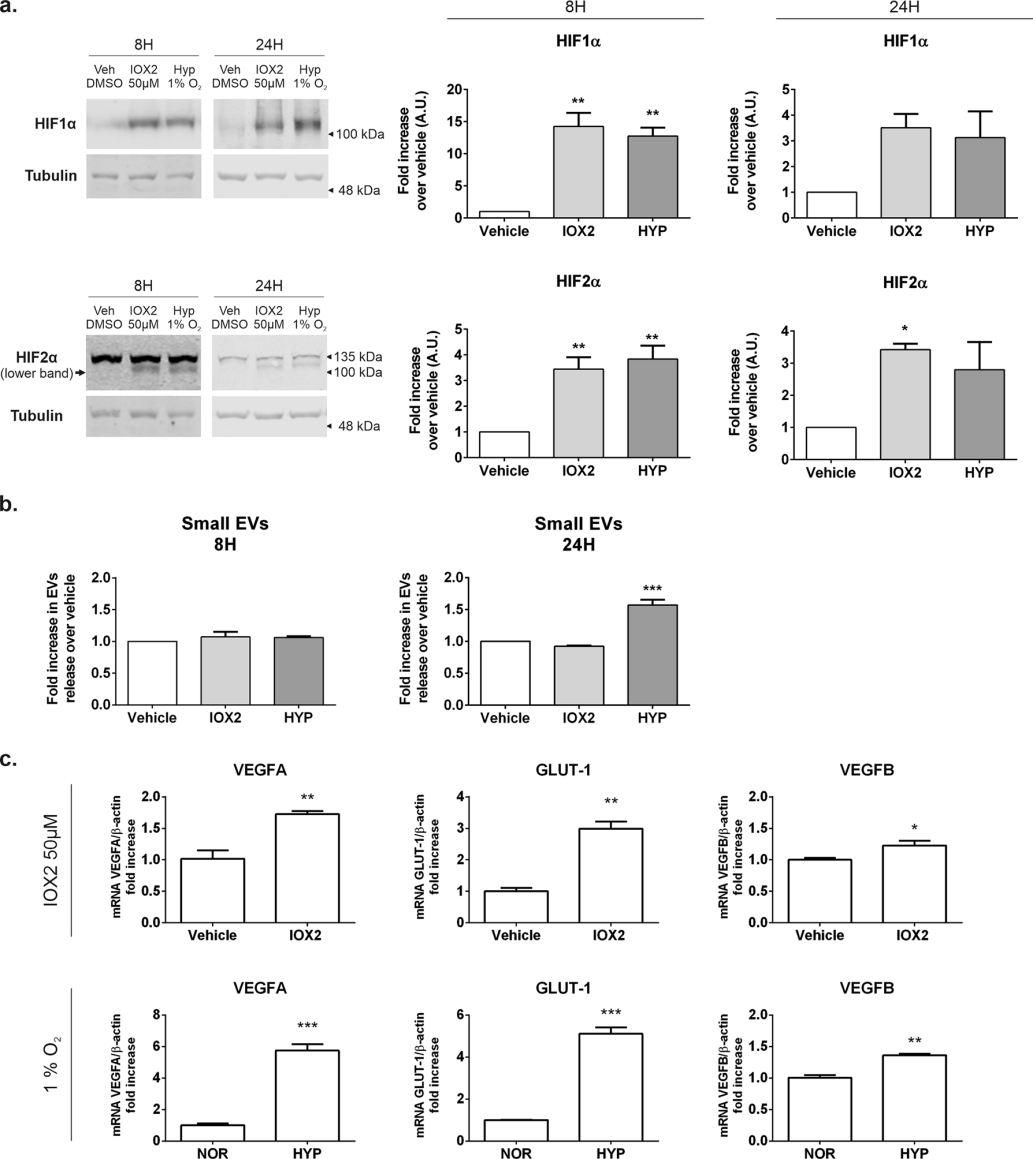
Prolyl hydroxylase inhibition does not increase small EV release in normoxic HEK293T cells. Expression of HIF1α and HIF2α in cells treated with vehicle (0.1% DMSO) or with 50 μM IOX2 for 8 h or 24 h. Cells were either left untreated or treated with IOX2 for the indicated times. (**a**) Cellular lysates were obtained, separated by SDS-PAGE and immunoblotted with specific antibodies for HIF1α, HIF2α or β-tubulin as loading control. Lower panels show the quantification of N = 3 independent experiments, each performed in triplicate. (**b**) Small EV release in cells treated with IOX2. Quantitation of small EVs by NTA measurement directly from the supernatant of the 10,000×*g* centrifugation of the conditioned media. Samples were conditioned media from cells either left untreated or treated with IOX2. Graphs show the amount of EVs corrected by total protein in cellular lysates and normalised over the vehicle mean of each independent experiment. Graphs show mean ± SEM of N = 3 independent experiments, each performed in triplicate. Statistic analysis: One-way ANOVA followed by Dunnet’s multiple comparisons test. *indicates < 0.05, **p < 0.01, ***p < 0.001. (**c**) Transcriptional activation of HIFs in IOX2 and hypoxia treated cells. Cells were either left untreated or treated with with 50 μM IOX2 in normoxic conditions, or placed under 1% hypoxia for 24 h. Total RNA was extracted and mRNA for HIF target genes was quantified by qPCR as detailed in the methods section. VEGFA and SLC2A1 are genes regulated by HIFs whereas VEGFB is not and was used as negative control. Statistical analysis: One-way ANOVA followed by Dunnet’s multiple comparisons test. *indicates < 0.05, **p < 0.01, ***p < 0.001.

As defined in previous studies^29^, we incubated HEK293T cells in DMEM 2% FBS EV-free media containing 50 µM of IOX2 for 8 h or 24 h to fully assess the effects of the drug in our cell system and we compared it to the effects of hypoxia exposure. We elected to treat cells for 8 h, the time point where we observed the greatest HIFα stabilization for both HIF subunits and for 24 h, the time point with maximal EV release. A subset of control cells were exposed to vehicle (0.1% DMSO) and either kept in normoxia or exposed to hypoxia for the indicated period of time. EVs were then isolated from the conditioned media of untreated cells or cells treated with IOX2 and in parallel we determined the stabilization of HIF proteins by immunoblot of cellular lysates. An independent set of cells were either left untreated or treated with IOX2 and subsequently used for RNA extraction to determine the level of HIF transcriptional activity by monitoring mRNA levels of *SLC2A1* and *VEGFA.* As shown in Fig. 6, we observed a robust stabilization of both HIF1α and HIF2α following the treatment with IOX2 (Fig. 6a), which was accompanied by a significant increase in the mRNA content of *SLC2A1* and *VEGFA*, indicative of HIF transcriptional activation (Fig. 6c). The mRNA levels for these HIF targets were increased over those seen in cells treated with vehicle but were smaller when compared to the levels observed when cells were exposed to hypoxia (Fig. 6c). However, despite the increase in HIF stability and transactivation activity seen in IOX2 treated cells, we did not observe any effect on the amount of EVs released to the conditioned media in IOX2 treated cells compared to vehicle, neither at 8 h or 24 h timepoint (Fig. 6b). As expected, cells subject to hypoxia showed both HIF stabilization and an increase in EV release.

These data suggest that HIF stabilization and activation alone in normoxic conditions may not be sufficient to trigger the EV release.

### Hypoxia induced integrated stress response (IRS) and autophagy may contribute to EVs release HEK293 cells

Our data suggests that HIF1α is a main driver for the increase in small EV release under hypoxia in HEK293 but its activation is not sufficient to trigger EV release in normoxic conditions. We therefore hypothesized that other homeostatic cellular responses triggered by the lack of oxygen and metabolic stress may also influence EV release along or downstream of HIF. In this context, we examined the activation of two cellular processes known to be activated under hypoxic conditions, namely autophagy and integrated stress response (IRS). Autophagy is a process by which cytoplasmic organelles are degraded to remove defective structures or as a means of obtaining macromolecules for energy generation in conditions of nutrient starvation. It has been reported that autophagy triggers exosome release. Autophagy occurs during long term exposure to hypoxia^30^ as a protective mechanism to prevent ROS accumulation and cellular death^31^, with HIF1α regulation of BNIP3 expression^31,32^ a protein involved in autophagy^33^ although this has not seen in other cell types^30^.

**Figure 7:**
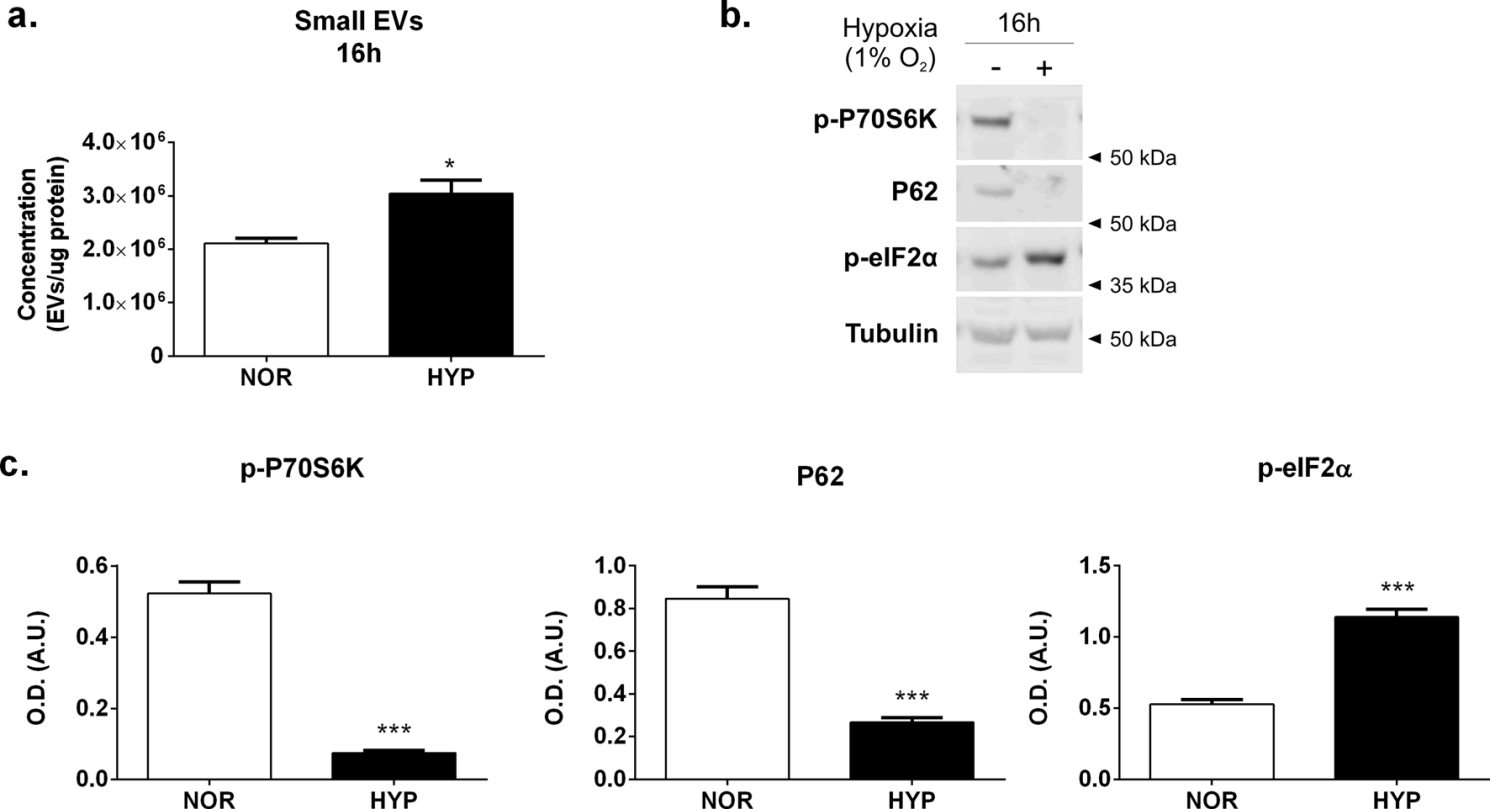
Induction of autophagy and Integrated Stress Response in HEK293T subject to hypoxia. Cells were cultured and either kept in normoxic conditions (NOR) or exposed to hypoxia (HYP) for 16 h in DMEM EV-free serum media. (**a**) Small EV release. Quantitation of small EVs by NTA measurement of vesicles in the supernatant of the 10,000×*g* centrifugation of the conditioned media. Graph shows the amount of EVs corrected by total protein content in cellular lysates. Graphs are mean ± SEM of n = 3 experiments, each measured in triplicate. Statistical analysis: Two tailed t-test, where *indicates p < 0.05. (**b**, **c**) Analysis of autophagy and ER stress/integrated stress response (ISR) markers. Cellular lysates were obtained from cells following 16 h of NOR/HYP exposure. Samples were resolved in SDS-PAGE and immunoblotted with specific antibodies against phospho-p70S6K at the Thr389 residue, p62, or phospho-eIF2α with β-tubulin used as loading control. (**b**) Representative blot and (**c**) Quantification of all the immunoblots carried out in Image J (NIH). Graphs show the mean ± SEM of N = 3. Statistical analysis: Two tailed t-test, where ***indicates p < 0.001.

To test our hypothesis we examined the activation of autophagy and IRS by means of immunoblotting of markers, including phosphorylation of p70S6 Kinase and p62 as general markers for autophagy and phosphorylation of eIF2α as a marker for PERK cascade activation in the ER stress/unfolded protein response (UPR). In this experiment, cells were either left in normoxic conditions or exposed to 1% hypoxia for 16 h, the time point at which we previously had detected a peak in EV release. Indeed, we reproduced an increase in small EV release in the conditioned media of the hypoxic cells compared to normoxic controls (Fig. 7a).

We then looked at the expression of markers for autophagy and UPR in the cellular lysates by immunoblotting. We found that the levels of phospho-p70S6K and p62 detected in the cellular lysates of cells exposed to hypoxia were significantly reduced as compared to the levels seen in cells kept in normoxic conditions (Fig. 7b,c). We also detected a robust phosphorylation of eIF2α indicating activation of ER stress response (Fig. 7b,c). These results suggest that both autophagy and ER stress /UPR are activated in HEK293 cells subjected to hypoxia and correlated with the peak of HIF activation and EV release in these cells^34,35^.

## Discussion

Extracellular vesicles carry bioactive molecules that serve as biomarkers for many human pathologies, some of the most studied perhaps being those arising from malignancies. Rapidly growing solid hypoxic tumours are known to produce large quantities of exosomes which influence the tumour microenvironment, promoting tumoral cell growth and survival^11,36–39^, immune avoidance and drug resistance^38,40^, and facilitate migration and metastasis^39,41,42^. Since hypoxia inducible transcription factors are key mediators of the metabolic and cellular adaptations to hypoxia, we tested the hypothesis that activation of the HIF pathway is responsible for enhancing EV release.

In our hands, the hypoxic stimulus increased the release of small extracellular vesicles in many cell types we tested, including fibroblasts, adipocytes, mesenchymal stem cells and human embryonic kidney cells. These findings are consistent with numerous studies in the literature supporting an increase in exosome release by cells exposed to low oxygen conditions^43^, including those released by hypoxic tumour cells^36,44–46^, and mesenchymal stromal cells^13^, transient hypoxia in the heart^47,48^ or cerebral ischaemia^49^. Mounting evidence also supports the notion that hypoxia not only stimulates EV release in cells but also induces changes in the composition of vesicles which direct impact on pathophysiology^43^.

However, we did not see significant changes in EV release in the cardiomyocyte cell line or in MDA-MB231 breast cancer cell line. It is possible that our hypoxic conditions (oxygen concentration and time) were not optimal to induce EV release in these cell types. In this regard, King et al.^50^ reported a small but significant 1.32 fold increase in EV release MDA-MB231 cells following exposure to hypoxia, albeit their exposure to hypoxia was much longer than in our study (48 h vs 24 h). The same authors also detected an increase in EV release in MCF7 cells (1.4 fold after 48 h) but not in the SKBR3 cancer cell line emphasizing the different response to hypoxia in breast cancer cells. The differences may reflect sutile different genetic makeups and/or tolerability to hypoxia. Indeed, hypoxia is known to influence the expression of thousands of genes^51,52^ predominantly regulated by HIF1^53^. However the extent of the transcriptional response to hypoxia is cell specific^52^.

Wang T et al., also detected an approximately twofold increase in the release of microvesicles in the MDA-MB231 cell line^54^. In this case the differences observed may be accounted for by the purification of different subsets of extracellular vesicles. While Wang et al. purified the vesicles at 10,000×*g* for 70 min, which enriches for lager, microvesicle type of vesicles,. our protocol included a further 100,000×*g* 75 min centrifugation from the 10,000×*g* supernatant, which results in a preparation of much smaller vesicles enriched in CD81 and thus, mostly of endosomal origin (exosomes). In any case, cells produce different types of vesicles all of which could be affected by exposure to hypoxia, and be affected by, both the length and severity of the hypoxic stimulus as well as by the nature of the cell type.

Our study showed HIF1α knockdown abolished the small EV release in hypoxic HEK293T cells, whereas no effect was seen with HIF2α knockdown, suggesting the effect on vesicle release in hypoxia is HIF1α dependent. In this regard, HIF1α overexpression increased exosomal release in cardiomyocytes^55^ and in bone marrow-derived mesenchymal stromal cells^13^. Similarly to our findings, King et al.^44^ showed that hypoxia increased exosome release in breast cancer cells was prevented by HIF-1α knockdown prior to hypoxic treatment. However, in our experiments, stabilization of the HIF pathway in normoxic conditions either by means of overexpression of oxygen-insensitive HIF1α mutant, or through a chemical inhibition of the repressor protein PHD2 did not have any effect on EV release in our cells. These findings indicated HIF1α stabilization in normoxia is not sufficient to enhance small EV release in our cells. It is possible that these results merely reflect a lower level of HIF1α activation achieved by these approaches to that obtained under hypoxic conditions, since for example, neither mHIF1α overexpression nor IOX2 treatment resulted in the same level of induction of VEGFA and GLUT1, two genes regulated by HIF, compared to the induction seen under hypoxia. Thus, it is possible that a threshold was not achieved for the expression of key molecules necessary for EV release. Another possibility is that the regulation of EV release under hypoxia may be a more complex process than that in normoxic conditions, whereby in hypoxia other cellular adaptive mechanisms besides HIF1α activation may also play an important role.

In this regard, while HIFs are master transcriptional regulators that drive the cellular adaptations and cell survival mechanisms under low oxygen conditions, the onset of hypoxia also activates additional molecular mechanisms, signalling pathways and cellular processes that could be involved in EV biogenesis and release. Among these, recent literature suggest that cellular events triggered secondary to the reduction in energy production caused by oxygen deprivation, including endoplasmic reticulum (ER) stress and activation of cellular autophagy may represent important mechanisms leading to EV formation and release. Kanemoto et al.^56^ reported ER stress enhanced multivesicular body formation and exosome release in cells following the treatment with tunicamycin. This was mediated through the IRE1 and PERK mediated pathways^56^. Autophagy, another cellular response mechanism activated downstream of metabolic stress that is also activated following ER stress^57–59^ has been associated with the release of exosomes^60,61^. In some cells autophagy is triggered following HIF activation via the accumulation of BNIP3. Consistent with all these findings, we observed a temporal correlation between the induction of the unfolded protein response (UPR) with the activation of the PERK pathway, with the onset of autophagy and the peak of EV release in our hypoxic HEK293T cells, occurring at 16 h post exposure to hypoxia. These data suggest these cellular processes may also be involved, however further experiments are required to elucidate the exact mechanism that cooperates with HIF1α activation and/or the molecular machinery downstream of HIF1α that is involved in this process.

While we have not determined the proteome or transcriptome profile of the vesicles produced by our hypoxic cells or by cells stably expressing mutant oxygen stable HIF proteins, these would give some hints on the nature of the biogenesis of the vesicles as well as the molecules that may be involved, and thus remain a subject for future studies. Nevertheless, it remains reasonable to speculate that HIF activation may regulate the composition of the hypoxic exosomes in our cells. Consistent with this hypothesis, a previous study overexpressing HIF1α in MSCs demonstrated changes in the cargo composition of both protein and miRNAs of exosomes produced by these cells^13^, which in turn had increased therapeutic properties^62^. Thus, it is tempting to speculate that HIF activation may contribute to regulate the expression or specific EV protein components or indeed regulate proteins that are involved in cargo selection or in vesicle biogenesis. Some emerging evidence suggest HIF upregulates the expression of Rab proteins, including Rab22a^12^ and Rab27a^46^, both of which have been associated with biogenesis and secretion of exosomes. The precise mechanisms by which hypoxia changes the composition of EVs remains largely unknown and deserves further exploration.

## Conclusion

Our results demonstrate that HIF1α activation is necessary to enhance small EV release in hypoxic HEK293 cells. However, our findings do not exclude the possibility that other cellular mechanisms that are upregulated during hypoxic conditions, may act in conjunction or downstream of HIF to regulate EV release. The precise molecular mechanisms underpinning the increase in EV release remain to be elucidated, given the role of EVs in human disease, this is an important question that warrants further investigation.

## Supplementary Information

Supplementary Information.

## Abbreviations

EVs: Extracellular vesicles
HIFs: Hypoxia inducible factors
MVB: Multi vesicular bodies
NTA: Nanoparticle tracking analysis
PHD2: Prolyl hydroxylase 2
VHL: Von Hippel–Lindau
