# Supplementary material for: Hypoxia-induced HIF1α activation regulates small extracellular vesicle release in human embryonic kidney cells
Source: Sci Rep. 2022 Jan 27;12:1443. doi: 10.1038/s41598-022-05161-7 (PMC8795438; doi:10.1038/s41598-022-05161-7)
Supplement: Supplementary file 1 — Supplementary Information. [file 41598_2022_5161_MOESM1_ESM.pdf]

Supplementary Information

Hypoxia-induced HIF1α Activation Regulates Small Extracellular Vesicle Release in Human Embryonic Kidney Cells.

Ana Muñiz-García, Montserrat Romero, Juan Manuel Falcón-Perez, Patricia Murray, Antonio Zorzano and Silvia Mora*

*Corresponding author: Dr. Silvia Mora, Universitat de Barcelona
Email: smora@ub.edu;

Supplementary Figures:

Figure S1

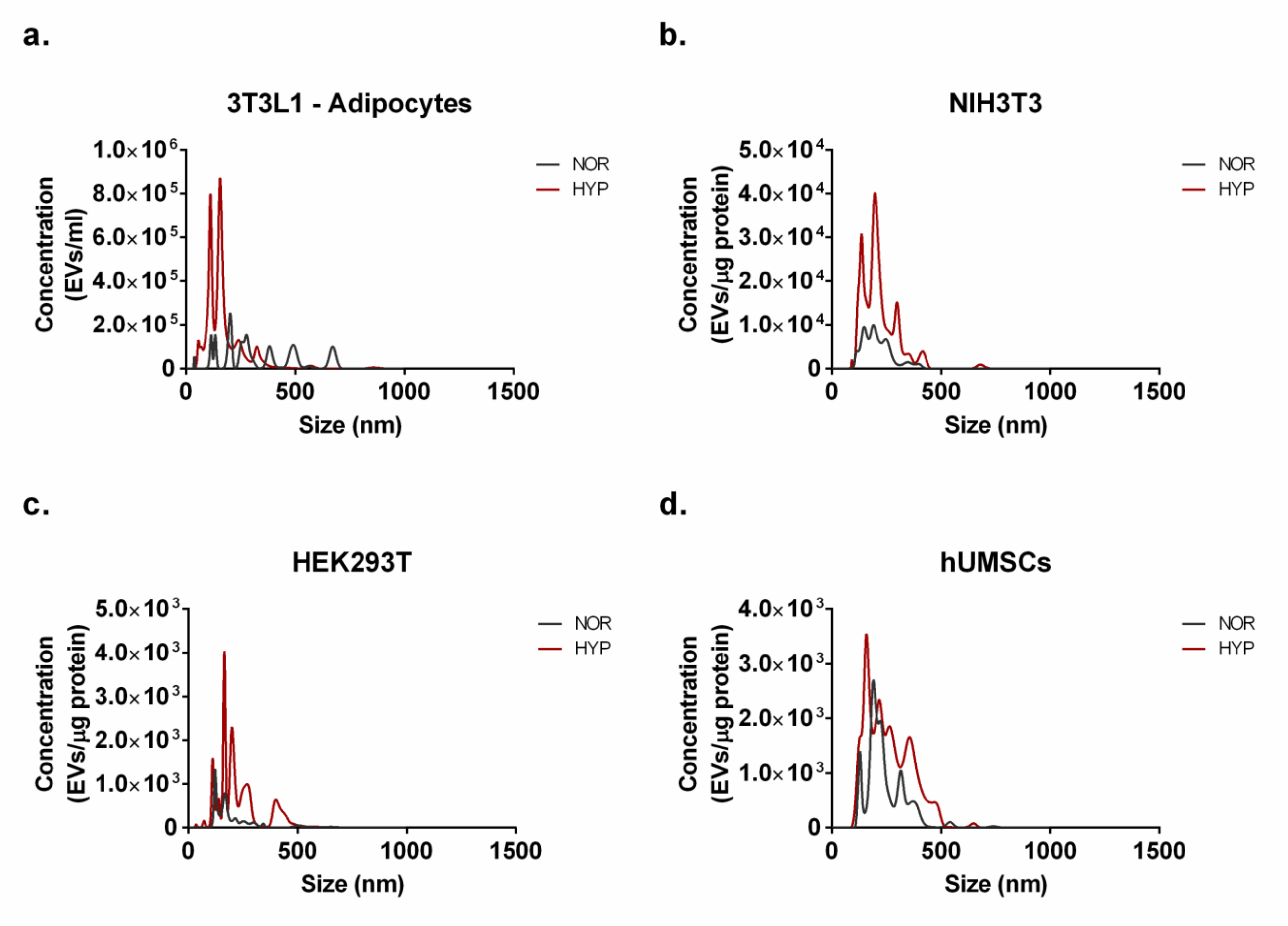

Supplementary Figure S1: Size distribution graphs of EVs obtained from a. 3T3L1, b. NIH3T3, c. HEK293T and d. hUMSC cells exposed to normoxia (NOR) or hypoxia (HYP). Data corresponds to the experiments shown in FIGURE 1. EVs were obtained by differential centrifugation as described in the methods section and obtained after a 100,000 xg ultracentrifugation step.

Figure S2

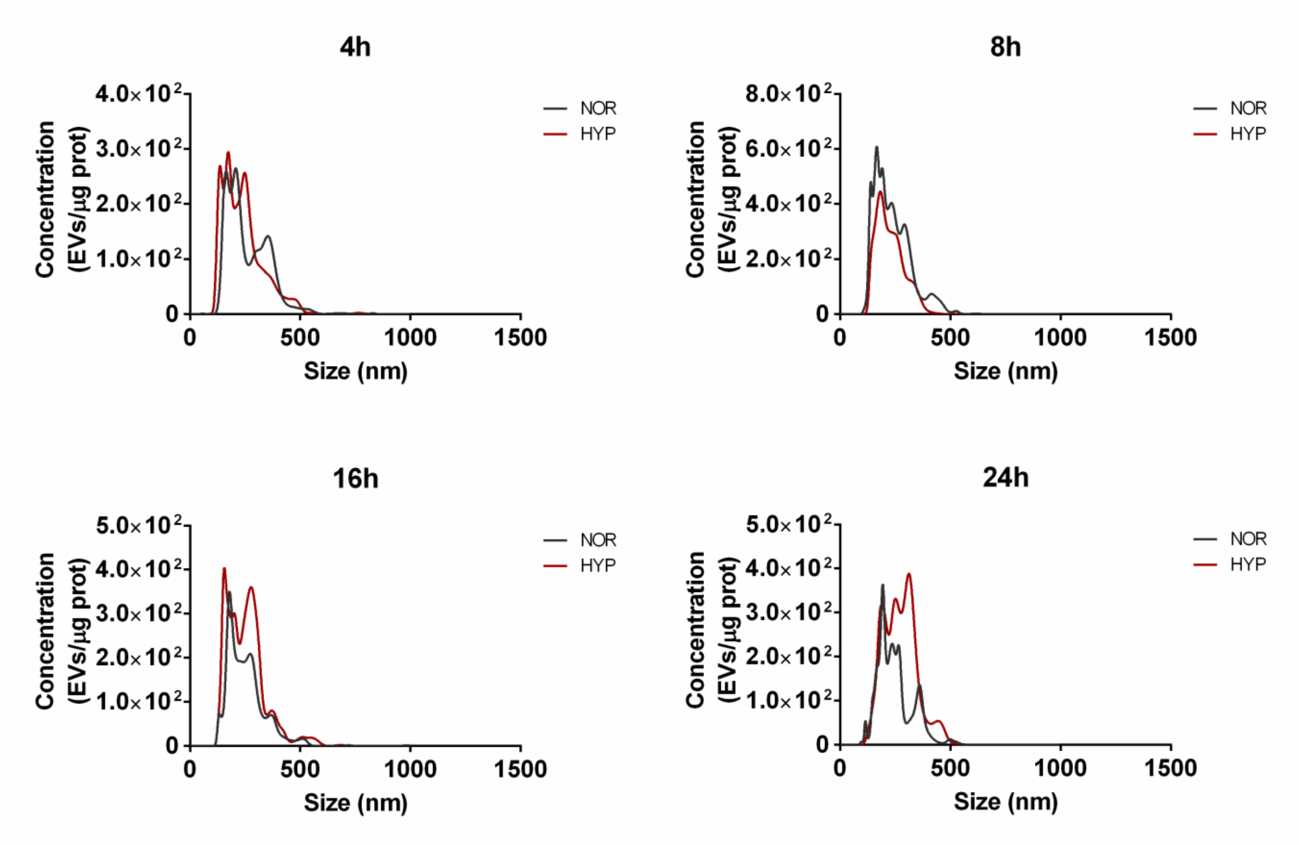

Supplementary Figure S2: Size distribution graphs of EVs obtained from HEK293T cells exposed to normoxia (NOR) or hypoxia (HYP) for different periods of time. Data corresponds to experiment shown in Figure 2. Graphs show the mean concentration for the different particle sizes normalised to the protein concentration of each sample's whole cell lysates obtained by the end of the experiment. EVs were obtained by differential centrifugation after an ultracentrifugation at 100,000 xg for 75 min.

Figure S3

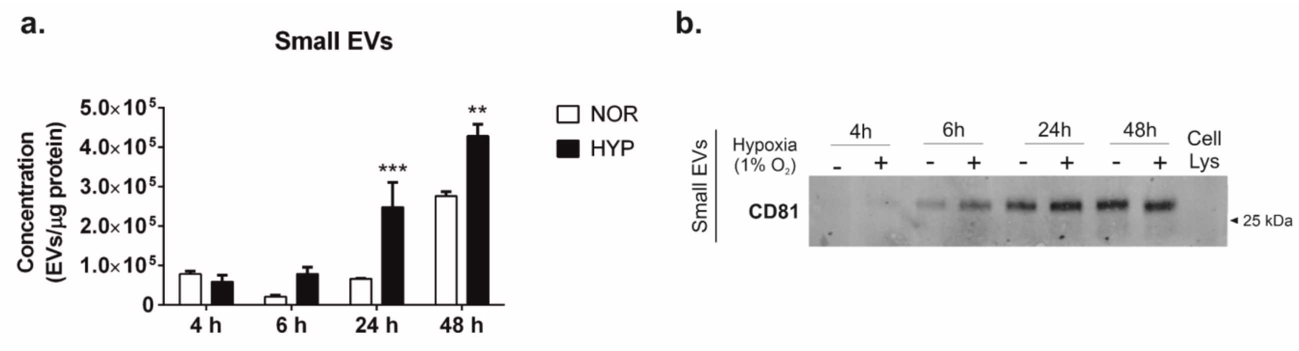

Figure S3: Partial EV charaterisation in small EVs from HEK293T cells exposed to normoxia (NOR) or hypoxia (HYP). a. EV release profile on HEK293T cells obtained during the time course hypoxia study. EVs were obtained by differential centrifugation protocol after an ultracentrifugation at 100,000 xg for 75 min. EVs were quantified by NTA. Data shows mean± SEM of n=3 samples per data point. Statistical analysis: Two-way

ANOVA, followed by Sidak's multiple comparisons test, where ** indicates $p<0.01$, and *** $p<0.001$. **b.** Immunoblot of EVs samples. Equal amounts (2µg) of EVS were resolved in SDS-PAGE and immunoblotted with an anti-CD81 antibody. 2µg of whole cell lysate was also loaded to confirm enrichment of the exosomal marker.

Figure S4

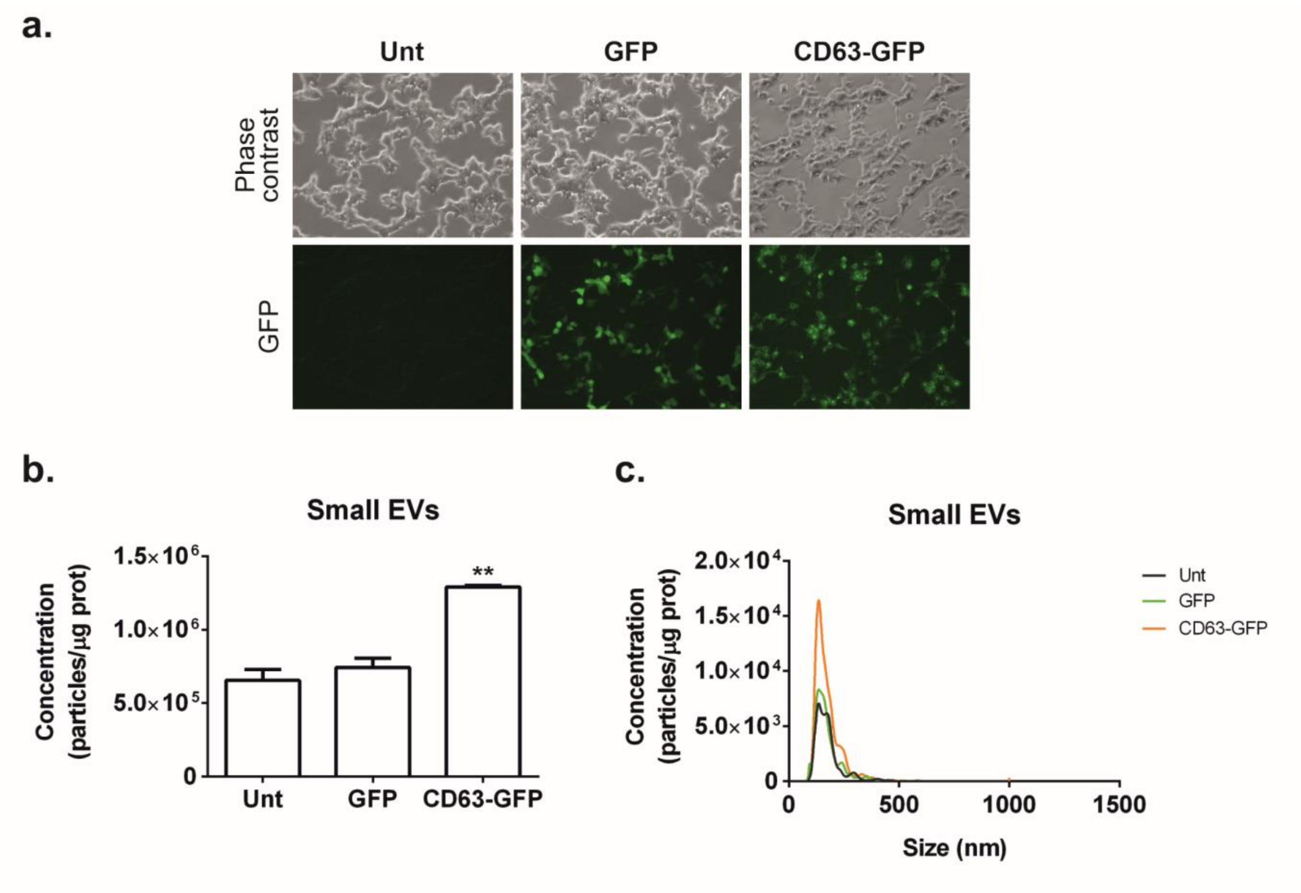

Figure S4: Validation of the detection of EVs release when measured directly from the conditioned medium by NTA from the 10,000 xg centrifugation, and its assessment of compatibility with Lipofectamine™ 3000 transfections. a. For this experiment HEK293T cells were transfected with a plasmid coding for the tetraspanin CD63 tagged with GFP (Addgene, #62964) using Lipofectamine 3000 following the manufacturers' instructions. Untransfected cells and cells transfected with the GFP expressing plasmid (Addgene, #54759) were used as controls. **b. and c.** Small EVs were quantitated and size distribution measured by NTA analysis from the supernatant after the centrifugation at 10,000 xg **b.** Graph show NTA data of the mean (± SEM) of released EVs measured by NTA and normalised to their respective total cellular lysate protein concentration. N=2 analysed by NTA in triplicate. Statistical analysis: One-way ANOVA, followed by Dunnett's multiple comparisons test. ** indicates $p<0.01$. **c.** Graph shows the mean size distribution of N=2 analysed by NTA in triplicate. **c**. Shows size distribution of isolated EVs.

Figure S5

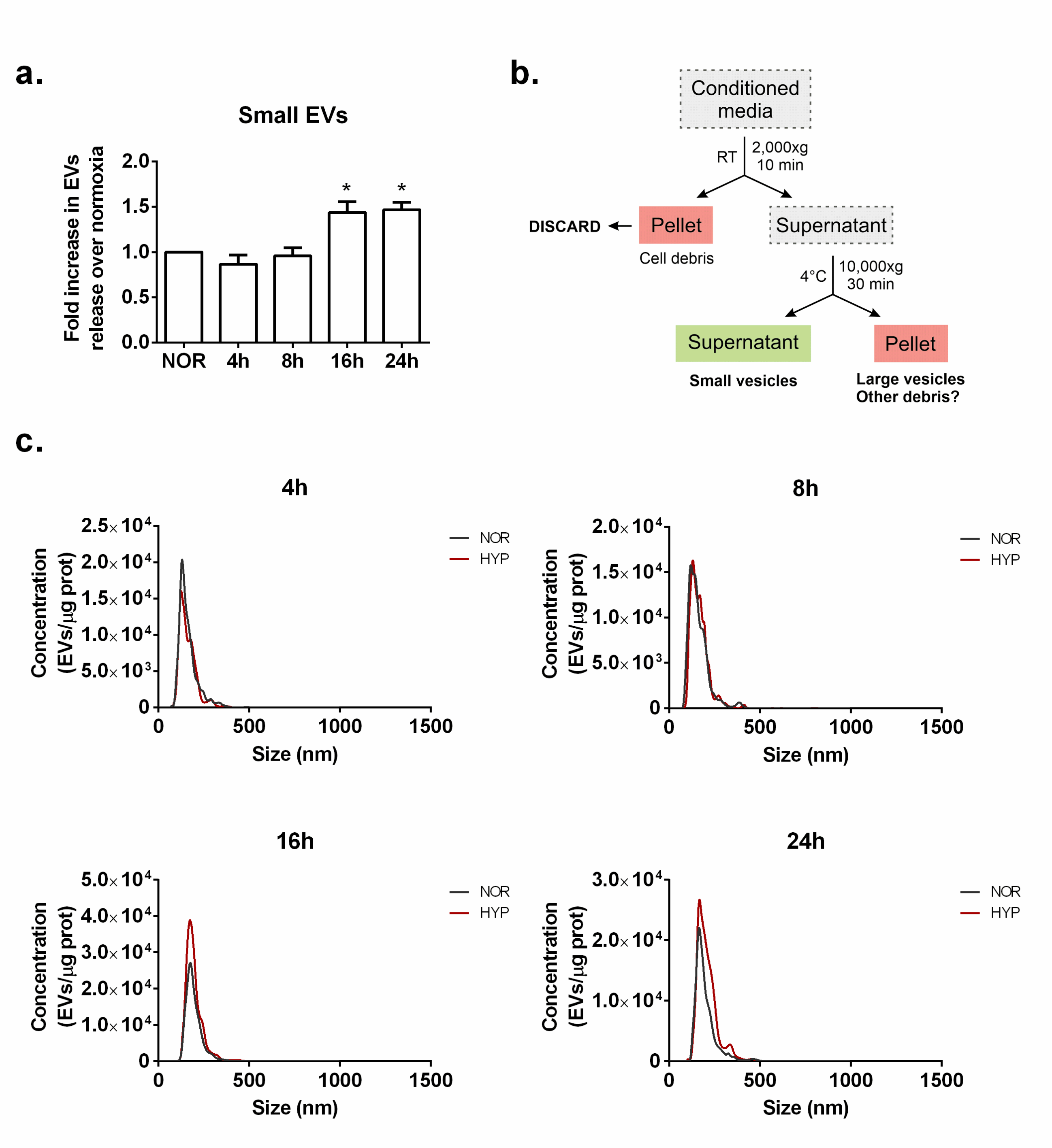

Figure S5: Evaluation of EV release from the conditioned medium in HEK293T cells exposed to normoxia (NOR) or hypoxia (HYP) for different periods of time. a. NTA analysis of small EVs released to the conditioned medium during the hypoxia time course. Graphs show mean (±SEM) of n=3 replicates. Data is normalised both to the protein concentration of their respective cellular lysates and to the EV release values of the normoxic condition of each time point. Statistical analysis: One-way ANOVA, followed by Dunnett's multiple comparisons test. * indicates $p<0.05$. **b.** Graphic representation of the protocol used to detect small EVs in the conditioned media. NTA was done on the supernatant following the 10,000 xg centrifugation. **c.** Size distribution graphs of the different time point studies showing NOR and HYP data at each time point. Graphs show mean concentration from n=3 normalised to the protein concentration of their respective whole cell lysates.

.

Figure S6

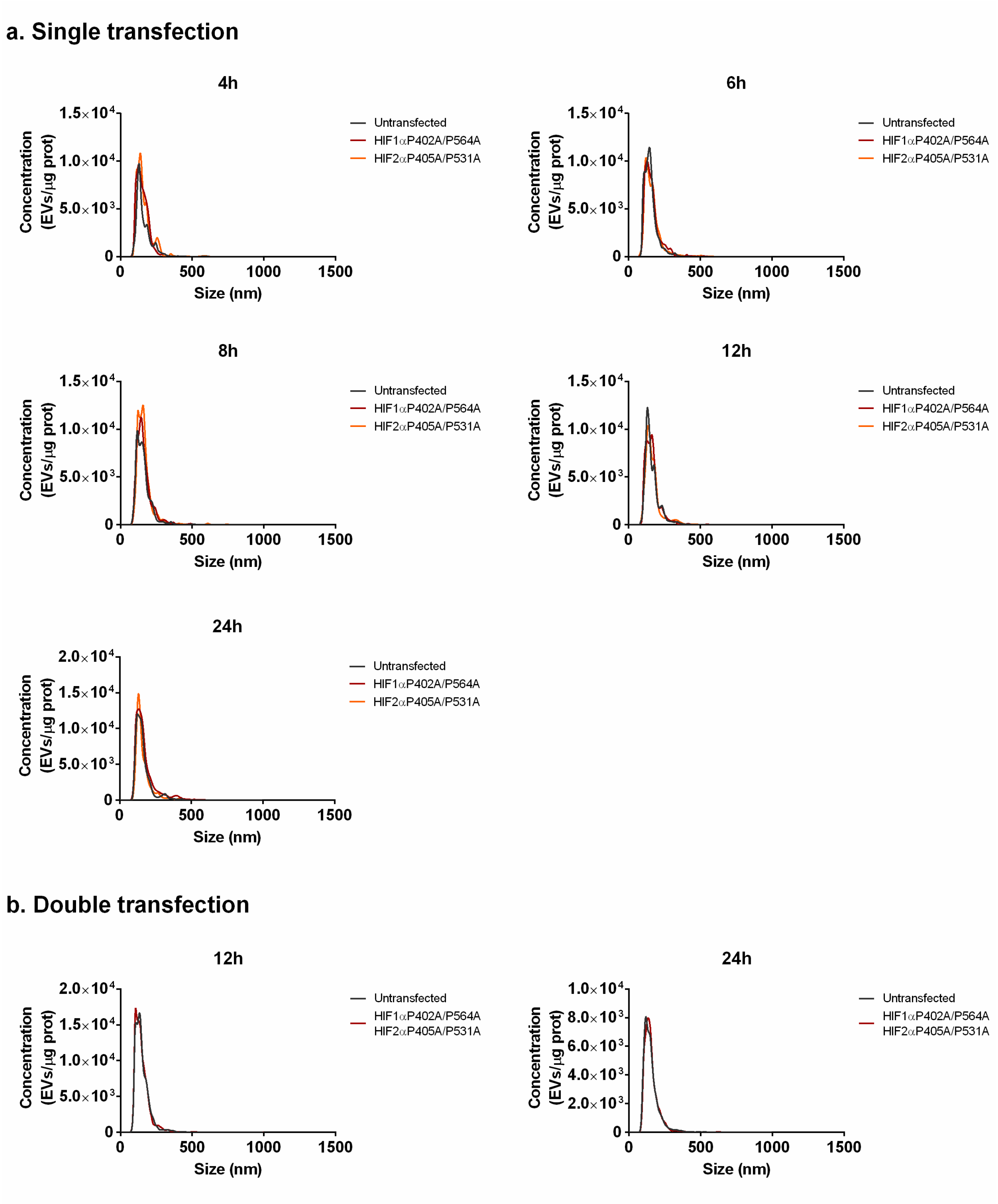

Figure S6: Size distribution graphs of EVs obtained from HEK293T cells expressing oxygen stable mHIF1α and/or m HIF2α. Graphs correspond to the data shown in Figure 5. Graphs show the mean concentration of all replicates for the different particle sizes normalised to the total protein concentration of their respective whole cell lysates. EVs were obtained by direct NTA measurement of conditioned media after the 10,000 xg

centrigugation. **a.** corresponds to the data from single transfection experiments **b**. shows data from double mHIF transfections, at different periods of time.

Figure S7

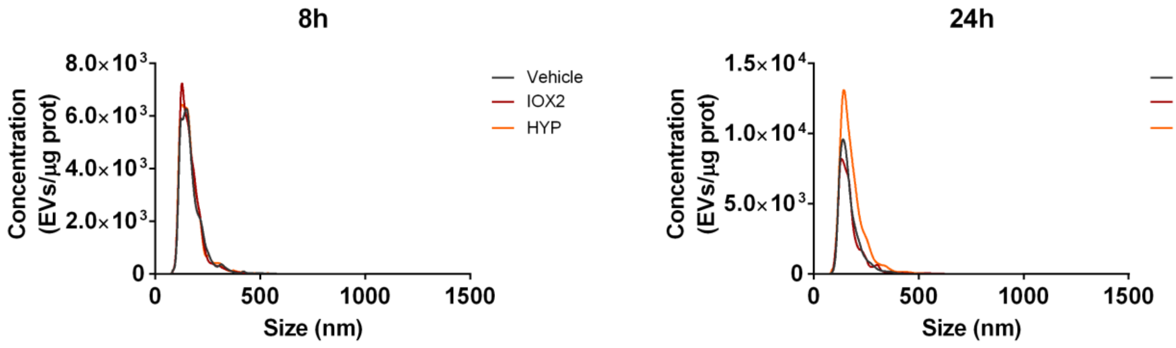

Figure S7: Size distribution graphs of EVs obtained from HEK293T cells treated with 50 µM of IOX2 compared to those exposed to hypoxia or untreated. Graphs correspond to the data presented in Figure 6**.** Graphs show the mean concentration of all replicates for the different particle sizes normalised to the protein concentration of their respective whole cell lysates at different periods of time. EVs size distribution was obtained by direct NTA measurement from the supernatant at the 10,000xg centrifugation.

Figure S8

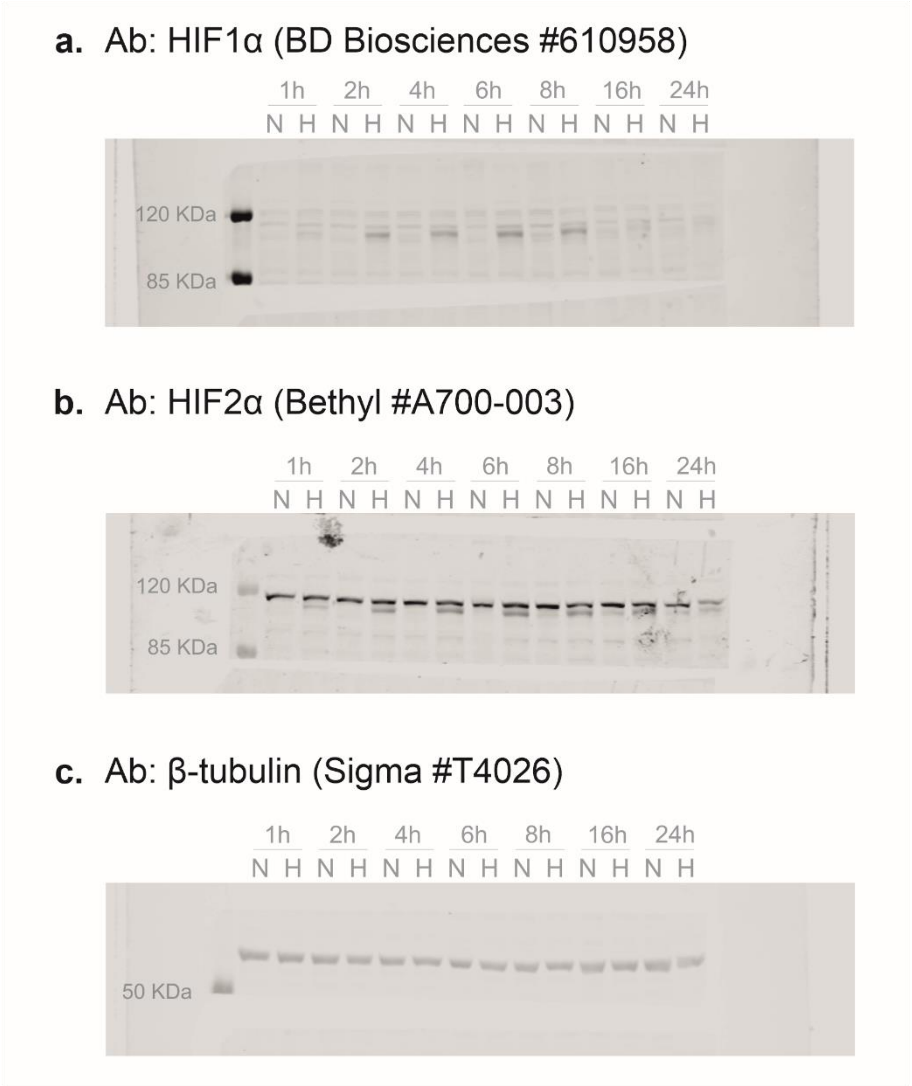

Figure S8: Original images from the western blot data shown in Figure 3A. Original images as acquired using LI-COR system. Image shows a representative nitrocellulose membrane cut after transfection below the molecular weight markers at 85kDa and 50KDa to probe different antibodies at a time: **a.** anti-HIF1α, **b.** anti-HIF2α and **c.** anti-β-tubulin. Gels were lab made at 10% acrylamide/bis-acrylamide 29:1 concentration. Shown ladder is Prestained Protein Molecular Weight Marker (#26612, ThermoFisher).

Figure S9

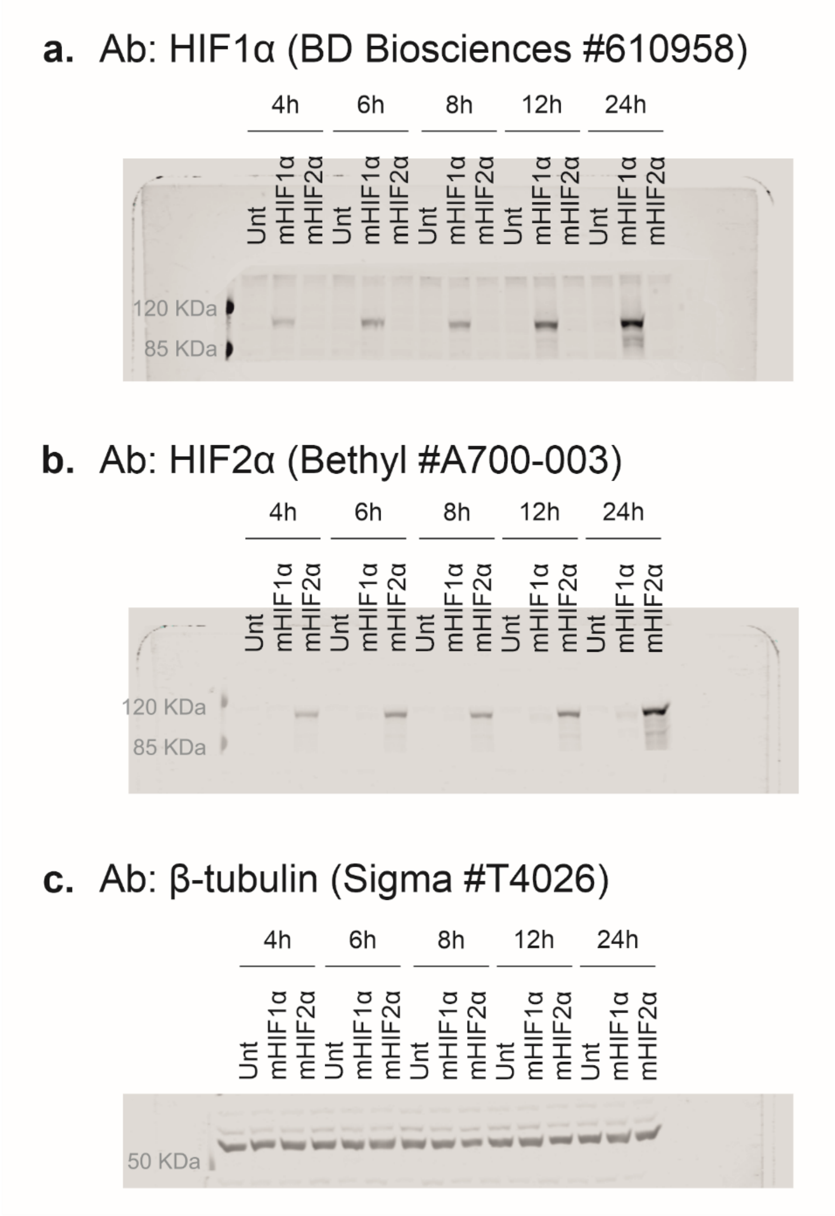

Figure S9: Original images from the western blot data shown in Figure 4A. Original images as acquired using LI-COR system. Image shows a representative nitrocellulose membrane cut after transfection below the molecular weight markers at 85KDa and 50kDa to probe different antibodies at a time: **a.** anti-HIF1α, **b.** anti-HIF2α and **c.** anti-β-tubulin. Gels were lab made at 10% acrylamide/bis-acrylamide 29:1 concentration. Shown ladder is Prestained Protein Molecular Weight Marker (#26612, ThermoFisher).

Figure S10

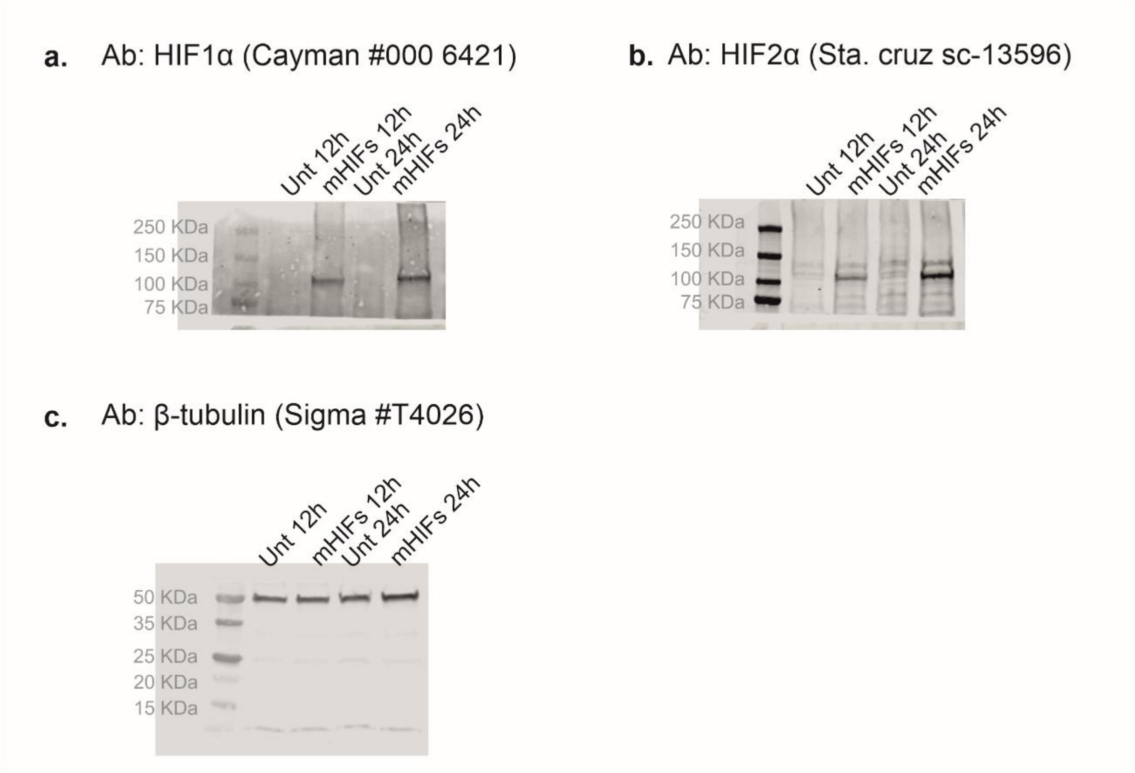

Figure S10: Original images from the western blot data shown in Figure 4C. Original images as acquired using LI-COR system. Image shows a representative nitrocellulose membrane cut after transfection below the molecular weight marker at 75KDa to probe different antibodies at a time: **a.** anti-HIF1α, **b.** anti-HIF2α and **c.** anti-β-tubulin. Gels were 4-20% Mini-PROTEAN® TGX™ Gel (#4561094, Bio-rad). Shown ladder is Precision Plus Protein™ All Blue Standards (#16610373, Bio-Rad).

Figure S11

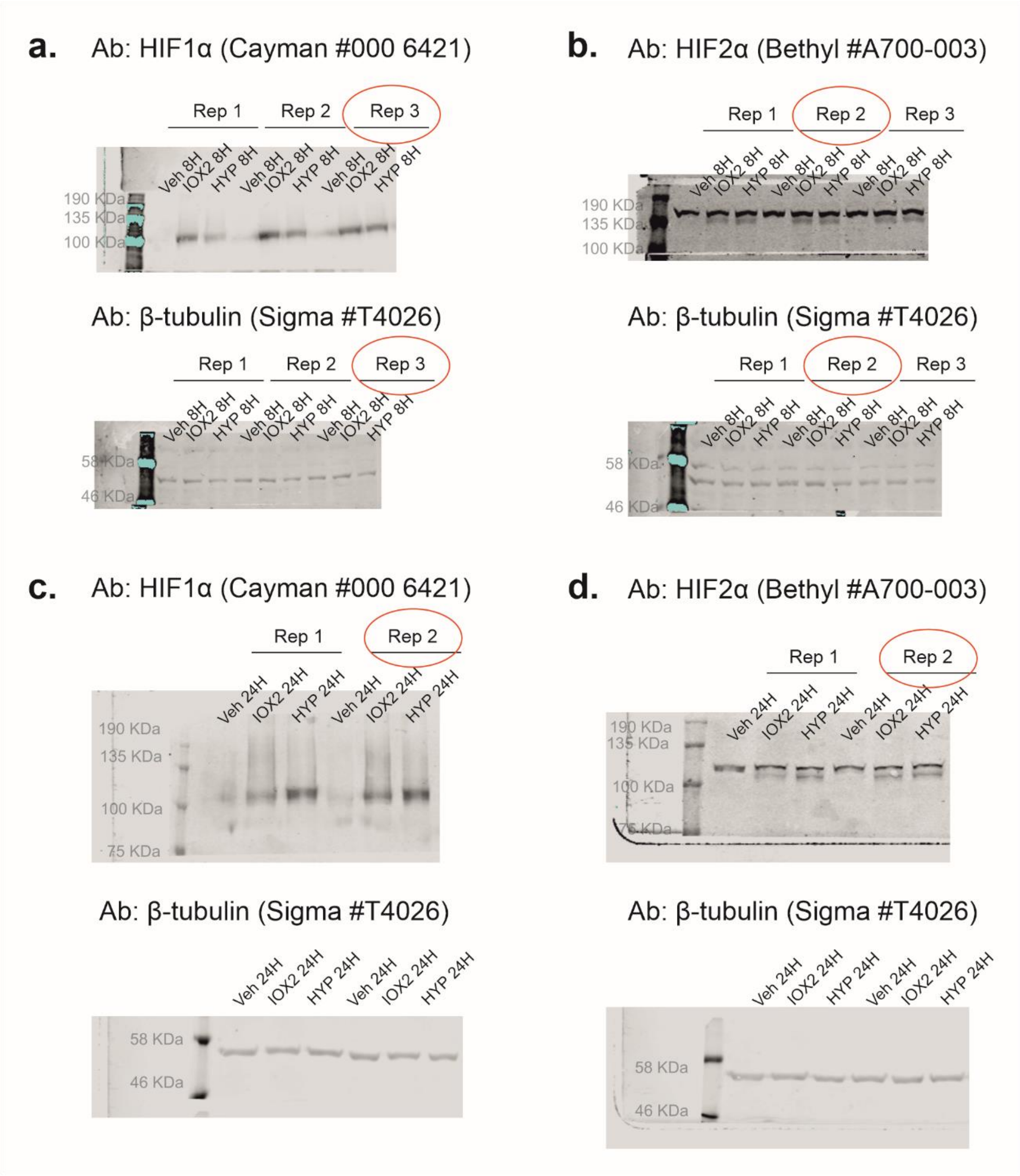

Figure S11: Original images from the western blot data shown in Figure 5A. Original images as acquired using LI-COR system. Image shows representative nitrocellulose membranes (red circled replicate is that shown in Figure 5A) cut after transfection below the molecular weight marker at 75KDa to probe different antibodies at a time: anti-HIF1α (**a.** and **c.**), anti-HIF2α (**b.** and **d.**) and anti-β-tubulin (**a., b., c.** and **d.**). Gels were lab made at 7 or 10% acrylamide/bis-acrylamide 29:1 concentration. Shown ladder is Blue Prestained Protein Standard, Broad Range (#P7706, NEB Biolabs).

Figure S12

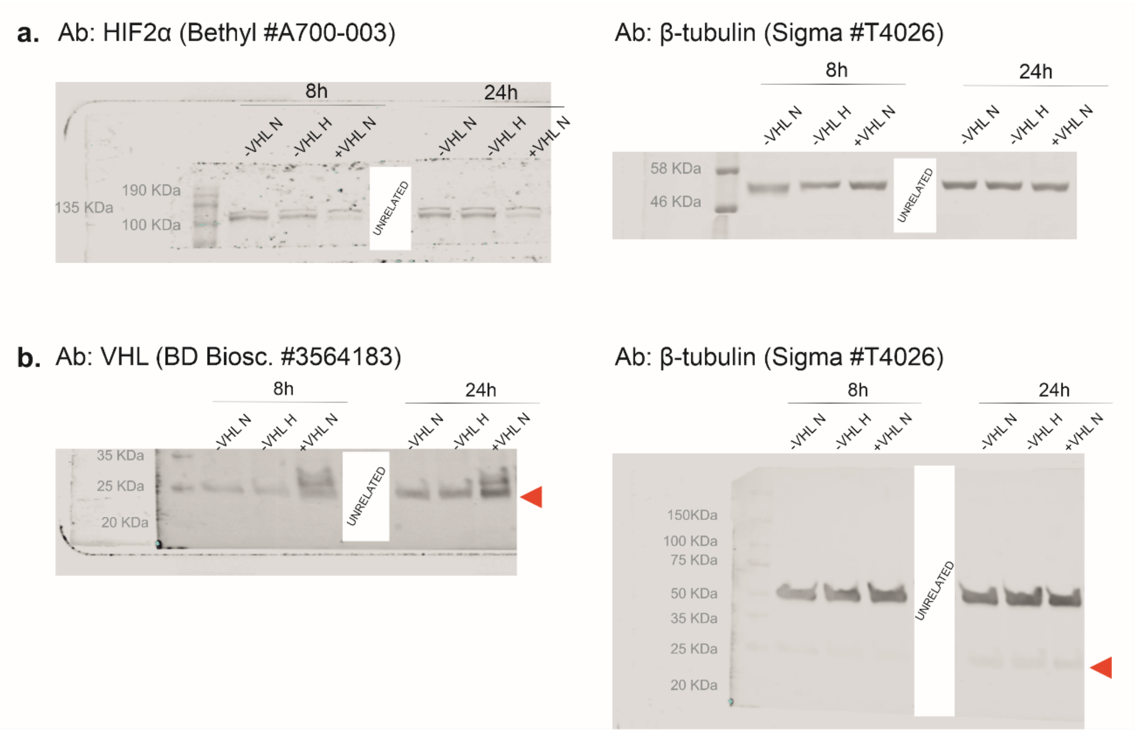

Figure S12: Original images from the western blot data shown in Figure 6A. Original images as acquired using LI-COR system. **a.** Image shows representative nitrocellulose membrane cut after transfection below the molecular weight markers at 75KDa and 46KDa to probe different antibodies at a time: anti-HIF2α and anti-β-tubulin. Gels were lab made at 10% acrylamide/bis-acrylamide 29:1 concentration. Shown ladder is Blue Prestained Protein Standard, Broad Range (#P7706, NEB Biolabs). **b.** Image shows representative nitrocellulose membrane immunostained with anti-VHL and anti-β-tubulin antibodies. Red arrow shows tubulin's unspecific band at 25KDa. VHL image acquisition stopped at 35KDa marker due to tubulin being in the same channel and its excessive brightness would affect VHL bands observation. Gel was 4-20% Mini-PROTEAN® TGX™ Gel (#4561094, Bio-Rad). Shown ladder is Precision Plus Protein™ All Blue Standards (#16610373, Bio-Rad).

Figure S13

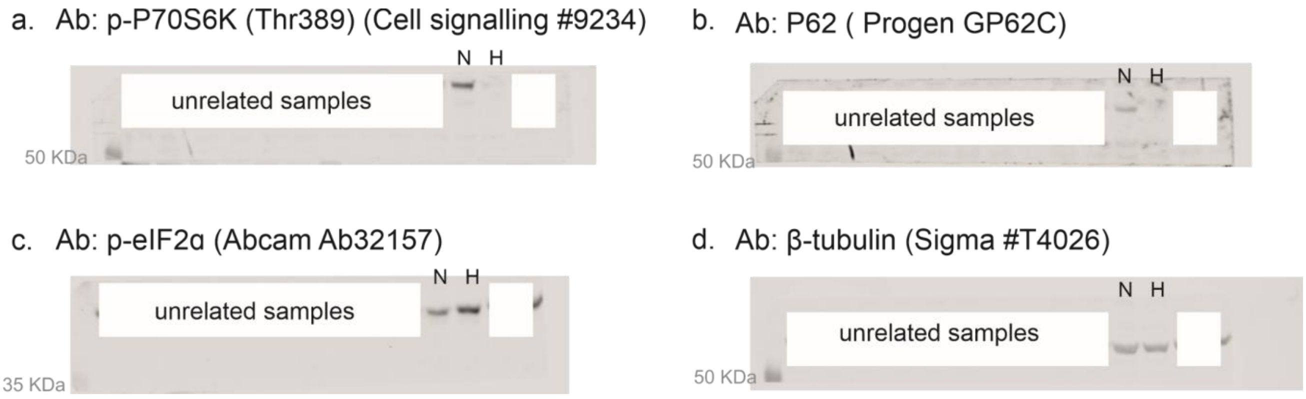

Figure S13: Original images from the western blot data shown in Figure 8B. Original images as acquired using LI-COR system. Image shows a representative nitrocellulose membrane cut after transfection below the molecular weight markers at 85KDa and 50KDa to probe different antibodies at a time: **a.** anti-phospho-P70S6K at Thr389 residue, **b.** anti-P62, **c.** anti-phospho-eIF2α and **d.** anti-β-tubulin. Gels were lab made at 10% acrylamide/bis-acrylamide 29:1 concentration. Shown ladder is Prestained Protein Molecular Weight Marker (#26612, ThermoFisher). (N=Normoxia, H= Hypoxia).
